# Targeting glutathione peroxidase 4 in ferroptosis: from immune regulation to pharmacological development and translational applications

**DOI:** 10.3389/fphar.2026.1834222

**Published:** 2026-08-27

**Authors:** Qianyue Yang, Yanfu Xia, Yonghe Wu, Liuting Zeng, Ganpeng Yu, Lingyun Sun

**Affiliations:** 1 Department of Rheumatology and Immunology, Nanjing Drum Tower Hospital Clinical College of Nanjing Medical University, Nanjing, Jiangsu, China; 2 Department of Pathology, Changde Hospital, Xiangya School of Medicine, Central South University (The First People’s Hospital of Changde City), Changde, Hunan, China; 3 Department of Obesity and Metabolic Diseases, Liling Hospital of Traditional Chinese Medicine, Liling, Hunan, China; 4 Department of Rheumatology and Immunology, Nanjing Drum Tower Hospital, Chinese Academy of Medical Sciences and Peking Union Medical College, Nanjing, Jiangsu, China; 5 Department of Orthopedics, People’s Hospital of Ningxiang City, Ningxiang, Hunan, China

**Keywords:** disease regulation, ferroptosis, glutathione peroxidase 4, lipid peroxidation, redox homeostasis, targeted therapy

## Abstract

Glutathione peroxidase 4 (GPX4) is a selenocysteine (Sec)-containing antioxidant enzyme and the only known mammalian enzyme capable of directly reducing membrane-embedded phospholipid and cholesterol hydroperoxides. It is recognized as a central regulator of ferroptosis, modulating cellular redox balance and influencing cell fate under oxidative stress. Despite a decade of research, critical gaps remain. Existing reviews largely focus on isolated diseases or single targeting strategies, and few provide an integrated framework that spans molecular regulation, physiological function, and clinical translation. The regulatory networks that control GPX4, from transcription to post-translational modifications and protein interactions, remain incompletely defined, and its ferroptosis-independent functions are underexplored. Moreover, the context-dependent and bidirectional roles of GPX4 across different diseases have not been systematically analyzed to guide appropriate therapeutic strategies. To address these gaps, this review delineates the structural basis and isoform-specific functions of GPX4, maps its multilayered regulatory network, and defines its roles across key physiological and pathological processes, including cancer, neurodegeneration, ischemia–reperfusion (I/R) injury, and autoimmune diseases. We also evaluate GPX4-targeted chemical strategies and analyze four core translational barriers: target specificity, systemic toxicity, acquired resistance, and tissue delivery, with evidence-based solutions for each. We conclude by identifying unresolved mechanistic questions and outlining priorities to accelerate clinical translation.

## Graphical Abstract

**Figure:**
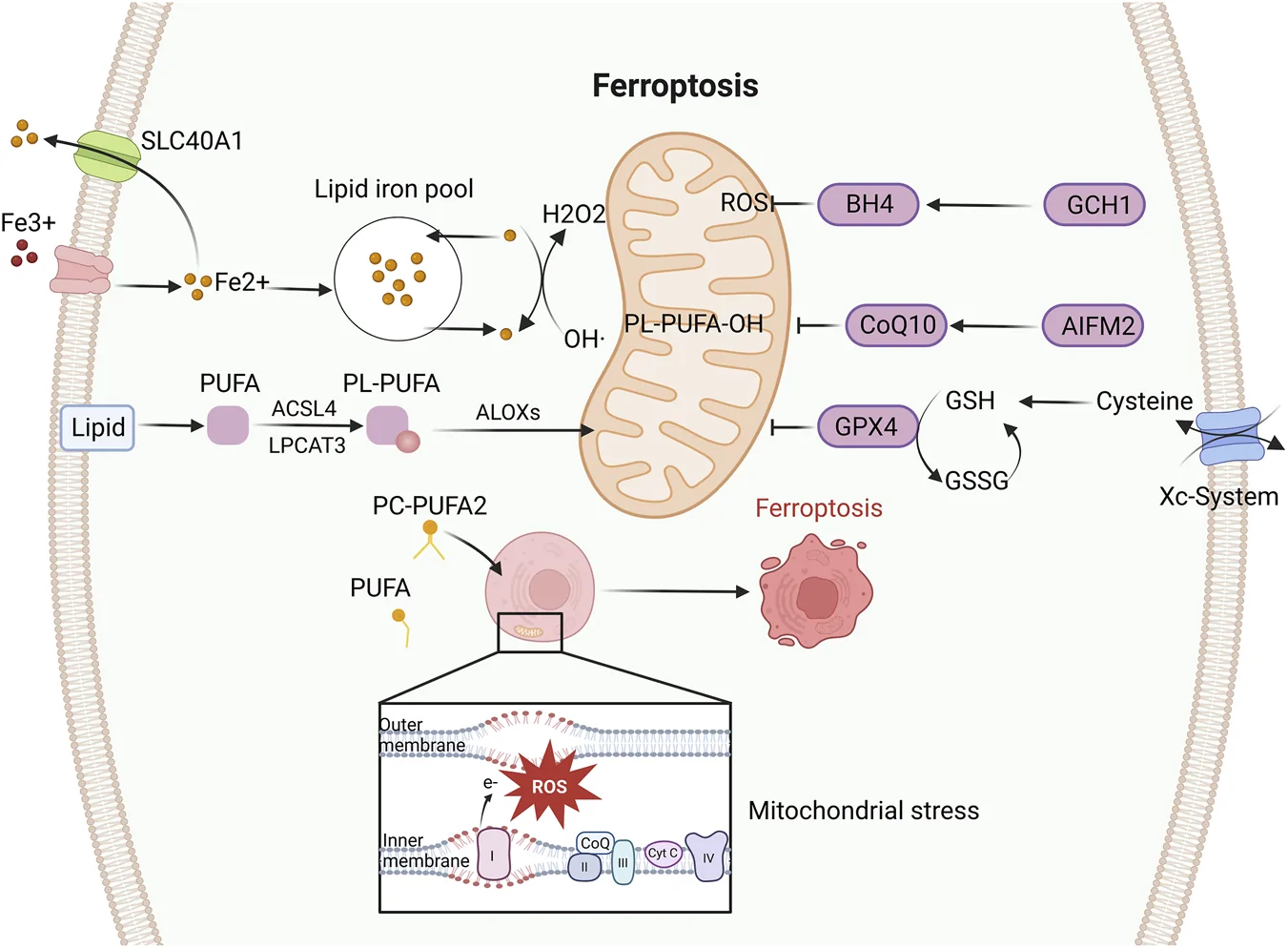

## Highlights

- This review connects glutathione peroxidase 4 (GPX4) biology to ferroptosis regulation across major human diseases.
- The multilayered regulatory network of GPX4 provides multiple nodes for pharmacological intervention.
- GPX4 is the only known enzyme that directly reduces membrane phospholipid hydroperoxides, a feature that distinguishes it from other antioxidant systems.
- Dysregulation of GPX4 occurs through transcriptional repression, post-translational modifications, and altered protein interactions. It has been linked to ferroptosis in cancer, neurodegeneration, organ injury, and autoimmune conditions.
- The context-dependent roles of GPX4 suggest that intervention strategies need to consider direction, timing, and cell-type specificity.

## 1 Introduction

The precise regulation of cell death is essential for organismal development, tissue homeostasis, and disease pathogenesis. Since ferroptosis was defined in 2012 as an iron-dependent, lipid peroxidation-driven process, its distinct features have expanded our understanding of cell death regulation and pointed to new therapeutic possibilities for refractory diseases, including cancer, neurodegenerative disorders, ischemia–reperfusion (I/R) injury, and autoimmune diseases (Qian et al., 2025a; Shahid et al., 2025; Wang et al., 2025a). Lipid peroxidation serves as the core execution mechanism of ferroptosis, and the clearance of membrane lipid hydroperoxides represents a critical checkpoint for cell fate (Lorenz et al., 2026).

Glutathione peroxidase 4 (GPX4) operates at this checkpoint. As a selenocysteine (Sec)-containing peroxidase, it is unique in its ability to directly reduce phospholipid and cholesterol hydroperoxides within biological membranes, a substrate preference that is largely absent in other GPX family members (Liu et al., 2025a; Xu et al., 2025a). Early studies showed that GPX4 functions both as an antioxidant enzyme and as a structural protein during sperm maturation (Tian et al., 2025). The subsequent framing of ferroptosis brought GPX4 to wider attention in redox biology and cell death research. Over the past decade, evidence has clarified its contributions to embryonic development, immune homeostasis, and cell differentiation, as well as its involvement in major human diseases (Park et al., 2025; Xu et al., 2025b). Manipulating GPX4 to modulate ferroptosis has been explored to overcome chemoresistance and radioresistance in cancer, to reduce neurodegenerative pathology, and to limit organ injury from ischemia–reperfusion (Zhao et al., 2025; Ye Y. et al., 2025).

Existing reviews generally address GPX4 in the context of a particular disease or therapeutic approach, and few integrate molecular mechanisms, regulatory networks, physiological functions, and clinical applications into one framework (Baskar, 2025; Lee et al., 2025). At the molecular level, findings on transcriptional regulation, post-translational modifications, and interacting partners remain fragmented, and a coherent picture of GPX4 control from transcription through protein–protein interactions (PPIs) is lacking (Huang et al., 2025; Chen Q. et al., 2025). Key unresolved questions include the distinct roles of GPX4 isoforms across subcellular compartments, the genetic mechanisms governing selenocysteine incorporation, the signaling routes that drive GPX4 degradation under pathological conditions, and the spatiotemporal behavior of its interaction network (Chen J. et al., 2025; Hu et al., 2025a). Addressing these issues is necessary for understanding how GPX4 dysfunction relates to disease and for achieving precise therapeutic targeting (Gu et al., 2025).

Functionally, the field has concentrated heavily on GPX4’s role in ferroptosis, whereas its ferroptosis-independent functions in spermatogenesis, immune cell homeostasis, erythroid differentiation, and lipid signaling remain underexplored (Na et al., 2025). Moreover, GPX4 displays bidirectional and cell-type-dependent effects across diseases. In many solid tumors, elevated GPX4 expression supports survival, metastasis, and therapy resistance (Cao et al., 2025a; Tang et al., 2025); in neurodegenerative conditions and acute organ injury, reduced GPX4 expression or activity drives cell death and tissue damage (Wu et al., 2025; Zhou et al., 2025a); and in autoimmune diseases, metabolic disorders, and chronic fibrosis, the reported roles are variable, with little systematic guidance on when and in which cell types to intervene (Liu et al., 2025b).

On the chemical side, covalent GPX4 inhibitors such as RSL3 have become standard research tools; however, most are restricted to basic studies, and clinical translation remains distant (Yu et al., 2025). These inhibitors often have limited selectivity, off-target engagement, unfavorable pharmacokinetics, and a narrow therapeutic window. Newer modalities such as proteolysis-targeting chimeras and molecular glues are only beginning to be applied to GPX4 (Yang et al., 2025). For approaches aimed at activating or stabilizing GPX4 in neuroprotection and tissue repair, selective candidates are scarce (Zhang et al., 2025a), and targeted delivery systems remain underdeveloped (Ji et al., 2026).

Translating these findings into clinical applications remains challenging. Systemic GPX4 inhibition causes on-target off-tissue toxicity as GPX4 is essential for embryonic development and somatic cell survival, leading to severe renal, intestinal, and neurotoxicity (Xiao et al., 2025). Tumor cells can acquire resistance through Nrf2 activation, upregulation of parallel ferroptosis defenses such as the FSP1–CoQ10 axis, and lipid metabolism reprogramming (Chaudhary et al., 2025). Clinically validated predictive biomarkers are lacking, and the long-term risks of GPX4 modulation have not been systematically evaluated.

In this study, we summarize recent advances in GPX4 research, covering its molecular biology, regulatory networks, physiology, disease roles, and therapeutic strategies. We review the structural basis and isoform-specific functions, map the regulatory network, outline dysfunction mechanisms in different pathologies, and evaluate chemical tools and their translational barriers, concluding with future directions.

## 2 Molecular biological characteristics of GPX4

### 2.1 Gene structure and transcript variants

The gene encoding human GPX4 is located in the p13.3 region of chromosome 19 (Grimwood et al., 2004). Recent large-scale genome-wide association studies have underscored the relevance of the GPX4 locus to human disease. In Crohn’s disease, the single-nucleotide polymorphism (SNP) rs7248137 at this locus has been identified as a susceptibility variant, reaching genome-wide significance (P = 2.1 × 10^−8^) and implicating GPX4 as a potential novel risk locus for inflammatory bowel disease (Franke et al., 2010).

The GPX4 gene has a complex structure and gives rise to at least three distinct protein isoforms through alternative splicing and the use of alternative promoters. These isoforms differ in their N-terminal sequences, which determine their subcellular localization and functions (Borchert et al., 2003). Large-scale full-length cDNA sequencing projects, such as the Mammalian Gene Collection initiative, have provided critical experimental evidence for elucidating the structure of genes with complex transcripts such as GPX4. By performing high-precision sequencing of cDNA libraries from diverse tissues, this initiative has provided validated cDNA clones containing the complete open reading frame for each gene, thereby overcoming the limitations of computational prediction based solely on genomic sequences and offering conclusive sequence evidence for the existence of distinct GPX4 splice variants (Gerhard et al., 2004; Strausberg et al., 2002).

Three major GPX4 isoforms have been characterized. The cytoplasmic isoform, c-GPX4, is the most extensively studied canonical form. It is translated from the second in-frame AUG initiation codon located in exon 2, and its N-terminus lacks the 27-amino-acid mitochondrial targeting signal present in m-GPX4, which accounts for its predominant cytoplasmic localization (Liang et al., 2009; Wang et al., 2003). This isoform is transcribed from an exon containing the AUG initiation codon and exerts a broad spectrum of antioxidant functions in the cytoplasm (Liang et al., 2009). Its N-terminal region contains a conserved HSC70-binding motif (residues 98–106) and a Ser104 phosphorylation site, both mediating its degradation through chaperone-mediated autophagy under oxidative stress (Wu et al., 2023; Zhu et al., 2025).

The mitochondrial isoform, m-GPX4, contains an additional upstream exon that encodes an N-terminal mitochondrial signal peptide of approximately 27 amino acids (Wang et al., 2003). This signal peptide directs the newly synthesized protein into the mitochondria, where it is processed to its mature form. The mature m-GPX4 is identical in size to c-GPX4 (Liang et al., 2009; Wang et al., 2003).

The nuclear or sperm-specific isoform, n-GPX4, is also referred to as sperm nucleus GPX. This isoform shows enriched expression in testicular tissue. Its unique N-terminal sequence is encoded by an alternative exon located within the first intron of the GPX4 gene. Transcription of this exon is regulated by an independent promoter containing binding sites for the cAMP response element modulator, which accounts for its stage-specific expression during spermatogenesis (Borchert et al., 2003; Tramer et al., 2004).

The existence of these transcript variants allows a single GPX4 gene to encode functionally compartmentalized proteins, enabling precise regulation of lipid peroxidation and redox signaling in distinct subcellular compartments.

### 2.2 Protein structure and catalytic mechanism

Unlike other GPX family members such as GPX1, which typically exist as tetramers, GPX4 is a monomeric enzyme. This structural feature is thought to be related to its ability to catalyze membrane-bound lipid substrates. The recently resolved crystal structure of human GPX4 reveals a compact globular fold, with the active site located in a relatively hydrophobic surface cleft that facilitates interaction with lipid membranes (Borchert et al., 2018).

The active site of GPX4 contains a highly conserved catalytic triad composed of selenocysteine, glutamine, and tryptophan. Selenocysteine is the catalytic center, and its unique chemical properties enable efficient peroxide reduction. The catalytic cycle of GPX4 follows a canonical ping-pong mechanism (Han et al., 2013). The selenocysteine residue in its reduced state is first oxidized by the peroxide substrate to form selenenic acid. This selenenic acid then reacts with the first molecule of reduced glutathione to form a selenenyl sulfide intermediate, with the release of one molecule of water. A second molecule of glutathione attacks this intermediate, regenerating the selenocysteine residue to its reduced state and producing oxidized glutathione (Xu et al., 2025b). The catalytic efficiency of GPX4 is therefore strictly dependent on intracellular glutathione availability, and its antioxidant function is severely impaired under conditions of glutathione depletion, such as that induced by the HIV-1 Tat protein (Choi et al., 2000).

The most distinctive biochemical feature of GPX4 is its substrate specificity. It is the only known enzyme that can directly reduce membrane-bound phospholipid hydroperoxides, cholesterol hydroperoxides, and other complex lipid hydroperoxides to their corresponding non-toxic alcohols (Schwarz et al., 2023). This function is essential for protecting cell membranes from lipid peroxidation damage. In this way, GPX4 directly regulates the intracellular peroxide tone, that is, the homeostatic level of lipid hydroperoxides. For example, GPX4 overexpression in the human breast cancer MCF-7 cell line reduces steady-state phospholipid hydroperoxide levels by more than 60%, indicating that GPX4 is a key determinant of the cellular redox environment (Wang et al., 2003). By contrast, other GPX isoforms mainly catalyze water-soluble small-molecule peroxides, such as hydrogen peroxide and short-chain fatty acid hydroperoxides (Schwarz et al., 2023). The ability of GPX4 to catalyze complex lipid substrates is thought to be related to its monomeric structure and the hydrophobic environment surrounding its active site, which may allow the enzyme to associate with the membrane surface and access peroxidized lipids within the bilayer (Borchert et al., 2018; Sutherland et al., 2001).

### 2.3 Subcellular localization and functional division

The distinct subcellular localization of GPX4 isoforms allows each to fulfill specific physiological roles in different compartments, creating a division of labor that has drawn considerable interest.

Mitochondrial GPX4 (m-GPX4) is a key component of the mitochondrial antioxidant defense system. Mitochondria are a major source of reactive oxygen species (ROS), and their inner membranes are rich in polyunsaturated fatty acids that are susceptible to oxidation. The presence of m-GPX4 is essential for maintaining the structural and functional integrity of mitochondria. Loss or inactivation of m-GPX4 leads to severe lipid peroxidation of mitochondrial membranes, disrupts the stability of electron transport chain complexes, impairs oxidative phosphorylation, and ultimately triggers cell death (Cole-Ezea et al., 2012). A recent study integrating subcellular fractionation, spatial proteomics, and mitochondrial importomics constructed a high-confidence human mitochondrial proteome. In this study, GPX4 was identified as one of more than 1,100 core mitochondrial proteins, with quantitative analysis confirming its abundance and turnover within the organelle (Morgenstern et al., 2021). Germline Gpx4 knockout in mice is embryonically lethal, and a transgene expressing cytoplasmic GPX4 can rescue this phenotype. Notably, this cytoplasmic isoform was found to partially localize to mitochondria, suggesting that the function of mitochondrial GPX4 is essential for mammalian survival (Boutet et al., 2009).

Cytoplasmic GPX4 (c-GPX4) is the most abundant isoform and is widely distributed in the cytoplasm. It protects the plasma membrane, endoplasmic reticulum membrane, and other organelle membranes from lipid peroxidation damage. This isoform is expressed in cells across all systemic tissues, including blood cells, and contributes to maintaining systemic redox homeostasis (Boutet et al., 2009). Beyond its antioxidant role, c-GPX4 participates in the regulation of signaling pathways. By controlling the levels of specific lipid peroxides, it influences inflammatory responses and cell death programs and also exerts a regulatory effect on cell proliferation. Lipid hydroperoxides themselves can act as signaling molecules involved in growth regulation, and GPX4 can function as a brake on cell proliferation by reducing their levels. In human breast cancer MCF-7 cells, GPX4 overexpression significantly prolongs cell doubling time and reduces plating efficiency, an effect mainly attributed to slowing the progression from the G1 to the S phase (Wang et al., 2003). This anti-proliferative effect appears to be specific as it does not induce compensatory changes in the expression of other major antioxidant enzymes, such as superoxide dismutase, catalase, or GPX1 (Wang et al., 2003).

Nuclear or sperm-specific GPX4 (n-GPX4) plays a dual role in male reproduction. During the late stages of spermatogenesis, n-GPX4 acts as a soluble, catalytically active peroxidase that protects the nucleus from oxidative damage, while chromatin undergoes extensive condensation (Ursini et al., 1999). After sperm maturation, however, its fate changes markedly. It loses enzymatic activity and polymerizes into insoluble proteins through oxidative cross-linking, becoming a major structural component of the mitochondrial capsule in the sperm midpiece, where it accounts for more than 50% of the total protein. This structural role is essential for maintaining the mechanical stability of the mitochondrial capsule and normal sperm motility. The midpiece structural defects and reduced motility observed in selenium deficiency-related male infertility are directly linked to impaired structural function of n-GPX4 (Ursini et al., 1999). Although its ultimate functions are associated with the sperm nucleus and mitochondria, immunoelectron microscopy studies have shown that n-GPX4 is predominantly localized in the cytoplasm in tissues such as the testis and kidney, suggesting that it may be transported to specific sites after synthesis (Borchert et al., 2003). With the continued development of protein interactomics, future studies are expected to further clarify the interaction networks and regulatory mechanisms of GPX4 isoforms in distinct subcellular compartments (Davis et al., 2015; Sowa et al., 2009; Wan et al., 2015).

## 3 Multi-level regulatory network governing GPX4 expression and activity

GPX4 abundance and activity are regulated at multiple levels, including transcription, translation, post-translational modifications, and metabolite-mediated mechanisms. This layered control enables cells to adjust lipid peroxide levels in response to developmental cues and environmental stress, thereby influencing ferroptosis susceptibility (Kumar et al., 2017).

### 3.1 Transcriptional regulation

The expression of the *GPX4* gene is regulated by multiple transcription factors and epigenetic mechanisms, with their roles varying across cell types and under stress conditions. The main regulatory factors are summarized in Table 1, grouped according to their effects on transcription.

**TABLE 1:** Summary of transcriptional regulators of GPX4.

Regulatory effect	Regulator/mechanism	Regulatory role and function	References (PMID)
Positive (transcriptional activation)	CREB/C/EBPα	Binds to the *GPX4* promoter to upregulate its expression. Promotes cell survival and inhibits ferroptosis during intestinal epithelial cell differentiation and in lung adenocarcinoma.	Speckmann et al. (2011) and Wang et al. (2021)
Positive (transcriptional activation)	Nrf2	As a core antioxidant transcription factor, it upregulates *GPX4* expression to resist ferroptosis. Exerts protective effects under multiple pathological conditions, including preeclampsia, viral infection, and acute injury.	Liao et al. (2022), Yuan et al. (2022), Shin et al. (2018), and S et al. (2023)
Positive (transcriptional activation)	Wnt/NR2F2	The Wnt signaling pathway activates NR2F2, which directly binds to the *GPX4* promoter to upregulate its expression, leading to chemoresistance in lung cancer brain metastasis cells.	Liu et al. (2021a)
Positive (transcriptional activation)	PITX2	Upregulates *GPX4* expression in pancreatic cancer cells to protect them from ferroptosis.	Wan et al. (2024)
Negative (transcriptional repression)	KLF Family (KLF2, KLF11, and KLF14)	Members including KLF2, KLF11, and KLF14 directly bind to the *GPX4* promoter and repress its transcription. Exerts tumor-suppressive effects and promotes ferroptosis in renal cancer, lung adenocarcinoma, and cervical cancer.	Lu et al. (2021), Zhao et al. (2023a), and Ye et al. (2024)
Negative (transcriptional repression)	IRF1	Downregulates *GPX4* expression through interaction with *SPI1* or induction by TNF-α, and promotes ferroptosis in colon cancer and renal allograft dysfunction.	Chen et al. (2023a) and Zhang et al. (2022)
Complex (predominantly negative)	p53	Can indirectly repress *GPX4* transcription. More importantly, p53 activates a unique ferroptosis pathway independent of GPX4 inhibition but dependent on *ALOX12*.	Cejas et al. (2007), Zhou et al. (2024a), Chu et al. (2019), and Chen et al. (2021a)
Negative (transcriptional repression)	CREMα	In systemic lupus erythematosus (SLE), it binds to the *GPX4* promoter and represses its transcription, leading to neutrophil ferroptosis and driving disease pathogenesis.	Li et al. (2021a)
Negative (transcriptional repression)	ERVW-1	In schizophrenia, it directly inhibits *GPX4* promoter activity to downregulate its expression, resulting in neuronal ferroptosis.	Zhang et al. (2024)
Negative (transcriptional repression)	TNFSF11 (RANKL)	Downregulates *GPX4* mRNA and protein levels in lung adenocarcinoma, thereby enhancing cellular sensitivity to ferroptosis inducers.	Li et al. (2024)
Negative (epigenetic repression)	DNA Methylation	Hypermethylation of the *GPX4* promoter region leads to its downregulated expression in chronic hepatitis B (CHB), which may facilitate viral immune evasion.	Su et al. (2024)

The expression of GPX4 is closely linked to the differentiation status of cells. This relationship is particularly evident in intestinal epithelial cells. Studies using Caco-2 cells as an *in vitro* model have shown that both GPX4 mRNA and protein levels increase substantially during differentiation and maturation. This upregulation is driven by enhanced promoter activity, with a composite binding site for the cAMP response element-binding protein (CREB) and the CCAAT/enhancer-binding protein (C/EBP) serving as the key cis-acting element responsive to differentiation signals. C/EBPα is considered a primary transcription factor regulating GPX4 expression in intestinal cells because its induced expression during differentiation correlates with GPX4 upregulation. Immunohistochemical analysis confirmed higher GPX4 expression in fully differentiated epithelial cells at the upper portions of small intestinal villi (Speckmann et al., 2011). In lung adenocarcinoma (LUAD), CREB has been shown to bind directly to the GPX4 promoter and upregulate its transcription, thereby inhibiting ferroptosis and promoting tumor cell survival and growth. This illustrates that the same transcription factor can serve opposite roles in normal differentiation *versus* malignancy (Wang et al., 2021).

As a transcription factor involved in the cellular antioxidant response, Nrf2 also participates in regulating GPX4 expression. In various cell types, Nrf2 activation upregulates antioxidant genes including GPX4 to counteract ferroptosis. For instance, in preeclampsia, the DJ-1 protein inhibits ferroptosis in trophoblast cells through the Nrf2/GPX4 axis (Liao et al., 2022). In Epstein–Barr virus (EBV)-infected nasopharyngeal carcinoma cells, the virus upregulates ferroptosis-resistance genes, such as GPX4 and SLC7A11, by activating the p62–Keap1–Nrf2 pathway, thereby enhancing resistance to ferroptosis (Yuan et al., 2022). Inhibition of the Nrf2 pathway can reverse the tolerance of head and neck cancer cells to GPX4 inhibitor-induced ferroptosis (Shin et al., 2018). In sepsis-induced acute lung injury, exosomes from adipose-derived stem cells deliver miR-125b-5p to inhibit Keap1, thereby activating Nrf2 and upregulating GPX4 transcription, which alleviates ferroptosis in pulmonary microvascular endothelial cells (S et al., 2023).

In the context of lung cancer brain metastasis, activation of the Wnt signaling pathway has been identified as a key upstream event driving GPX4 upregulation. The pathway activates the nuclear receptor family member NR2F2, which binds directly to the GPX4 promoter as a transcriptional activator. This Wnt/NR2F2/GPX4 axis is one of the core mechanisms contributing to acquired platinum-based chemoresistance in brain metastatic tumor cells (Liu W. et al., 2021). PITX2 has also been identified as a transcriptional activator of GPX4 that protects pancreatic cancer cells from ferroptosis (Wan et al., 2024).

Several transcriptional repressors exert tumor-suppressive effects by directly binding the GPX4 promoter. KLF2, KLF11, and KLF14 have all been shown to directly bind the GPX4 promoter and repress its transcription, thereby promoting ferroptosis and inhibiting tumor progression in renal, lung, and cervical cancers (Lu et al., 2021; Zhao G. et al., 2023; Ye et al., 2024). Among these, the role of KLF11 is particularly well defined as its downregulation in lung adenocarcinoma relieves GPX4 repression and drives chemoresistance (Zhao G. et al., 2023). Similarly, IRF1 represses GPX4 transcription, although its mode of action appears context-dependent. In colon cancer, it acts by interfering with the transcriptional activator SPI1 (Chen Y. et al., 2023). In chronic renal allograft dysfunction, its upregulation is driven by TNF-α signaling (Zhang et al., 2022).

The relationship between the tumor suppressor p53 and GPX4 is complex and context-dependent. On the one hand, p53 may indirectly downregulate GPX4 through transcriptional repression as GPX4 expression is negatively correlated with p53 accumulation in breast cancer tissues (Cejas et al., 2007). This negative regulatory mechanism has been further explored in acute liver failure, where activation of sirtuin 1 (SIRT1) leads to deacetylation and inactivation of p53, thereby relieving transcriptional repression of GPX4 and ultimately upregulating its expression to protect hepatocytes from ferroptosis (Zhou XN. et al., 2024). On the other hand, substantial evidence indicates that p53 can activate a pro-ferroptotic pathway independent of GPX4 inhibition. A landmark study showed that p53 activation (e.g., by Nutlin treatment) induces ferroptosis without affecting GPX4 enzymatic activity or causing glutathione depletion or alterations in the GSH/GSSG ratio. Through RNAi screening, arachidonate 12-lipoxygenase (ALOX12) was identified as an essential effector in p53-mediated ferroptosis. ALOX12 knockout completely abrogates p53-dependent ferroptosis and its tumor-suppressive function in xenograft models but does not affect ferroptosis induced by GPX4 inhibitors such as RSL3 (Chu et al., 2019). Additionally, p53 can promote ferroptosis by inhibiting iPLA2β, a phospholipase with antioxidant repair functions, further highlighting its diverse, GPX4-independent mechanisms (Chen D. et al., 2021).

In systemic lupus erythematosus (SLE), transcriptional repression of GPX4 is a core event leading to neutrophil ferroptosis and disease pathogenesis. Autoantibodies and interferon-α (IFN-α) in SLE serum act synergistically to enhance the binding of the transcriptional repressor cAMP-responsive element modulator α (CREMα) to the GPX4 promoter through specific signaling pathways. This increased binding significantly represses GPX4 transcription and expression, leading to lipid peroxide accumulation and triggering ferroptosis in neutrophils. This mechanism not only explains the neutropenia often observed in SLE patients but also highlights its role in autoimmunity. Notably, mice with neutrophil-specific Gpx4 haploinsufficiency recapitulate the key clinical features of human SLE (Li et al., 2021a).

In schizophrenia, the envelope protein gene of the human endogenous retrovirus W family (ERVW-1) has been identified as a transcriptional repressor of GPX4. Clinical data show that ERVW-1 expression is significantly negatively correlated with GPX4 and SLC3A2 expression in the brain tissue of schizophrenic patients. Mechanistic studies confirmed that ERVW-1 directly inhibits GPX4 promoter activity, downregulating its transcription and protein expression and leading to neuronal ferroptosis. This reveals a novel mechanism by which endogenous retroviruses contribute to psychiatric disorders through regulating a core ferroptosis gene (Zhang et al., 2024).

Tumor necrosis factor superfamily member 11 (TNFSF11), also known as RANKL, is another cytokine that regulates GPX4 expression. Its expression is significantly upregulated in lung adenocarcinoma. Mechanistic studies have found a negative correlation between TNFSF11 and GPX4 expression, with TNFSF11 overexpression reducing both GPX4 mRNA and protein levels. As a result, LUAD cells with high TNFSF11 expression exhibit greater sensitivity to ferroptosis inducers, such as erastin and RSL3. This suggests that cytokine-mediated GPX4 downregulation may be an important determinant of ferroptosis susceptibility in certain tumor types (Li et al., 2024).

**FIGURE 1:**
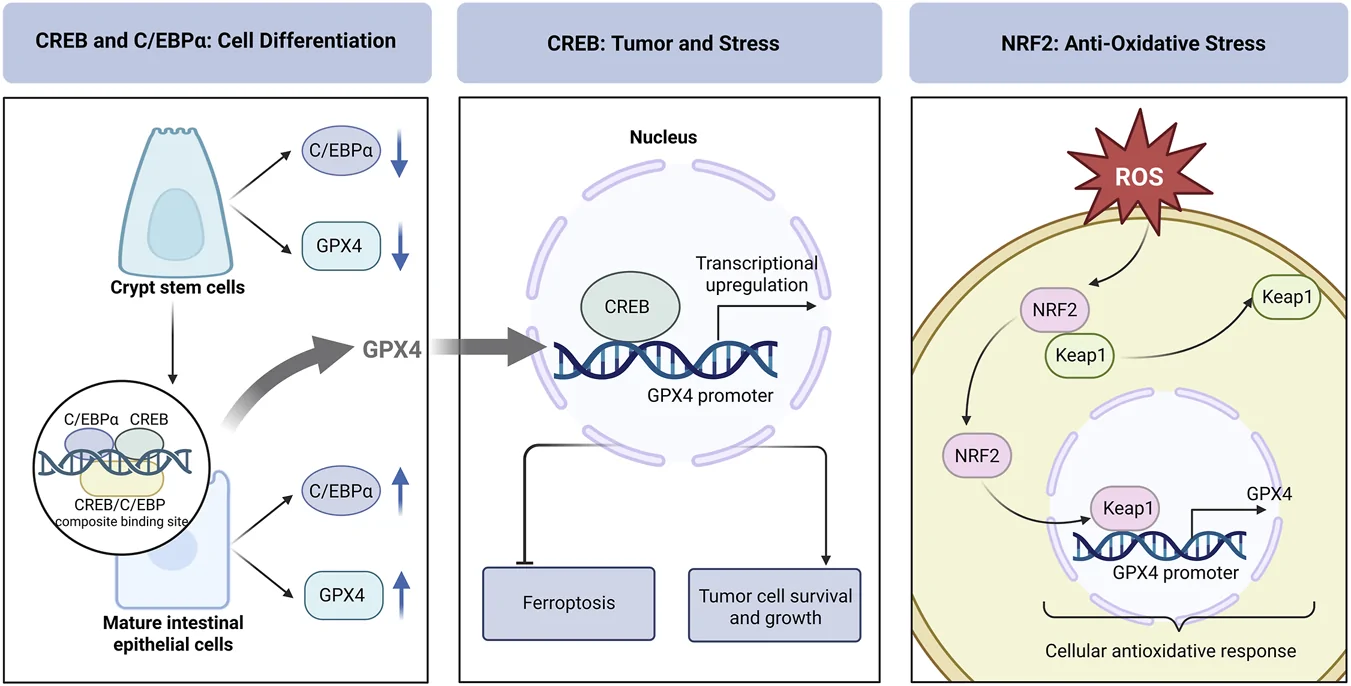
Core transcriptional regulatory axes of GPX4 in physiological and pathological processes. The CREB/C/EBPα axis drives GPX4 transcription during intestinal epithelial cell differentiation to maintain intestinal tissue homeostasis. In tumor cells, CREB directly upregulates GPX4 transcription to suppress ferroptosis and promote tumor growth. The canonical NRF2-Keap1 axis mediates GPX4 upregulation under oxidative stress via ARE binding in the GPX4 promoter, initiating cellular antioxidant responses and inhibiting ferroptosis. These pathways collectively govern GPX4 expression in a context-dependent manner, modulating ferroptosis in both physiological and pathological settings.

Beyond transcription factor-mediated regulation, GPX4 expression is also tightly controlled by epigenetic modifications, particularly DNA methylation in the promoter region. In patients with chronic hepatitis B (CHB), hypermethylation of the GPX4 promoter has been observed, most prominently during the immune tolerance phase. This hypermethylation correlates negatively with GPX4 mRNA and protein levels, indicating that promoter methylation serves as a key transcriptional repression mechanism in CHB. Functionally, this GPX4 downregulation is thought to inhibit the cGAS–STING antiviral immune pathway, thereby facilitating viral escape from immune surveillance and maintenance of immune tolerance (Su et al., 2024) [Figure 1].

The CREB/C/EBPα axis drives GPX4 transcription during intestinal epithelial cell differentiation to maintain intestinal tissue homeostasis. In tumor cells, CREB directly upregulates GPX4 transcription to suppress ferroptosis and promote tumor growth. The canonical NRF2–Keap1 axis mediates GPX4 upregulation under oxidative stress *via* ARE binding in the GPX4 promoter, initiating cellular antioxidant responses and inhibiting ferroptosis. These pathways collectively govern GPX4 expression in a context-dependent manner, modulating ferroptosis in both physiological and pathological settings.

### 3.2 Translational and post-translational regulation

The translational efficiency, protein stability, and enzymatic activity of GPX4 are precisely regulated through translational mechanisms, post-translational modifications, and associated regulatory pathways. Key regulatory mechanisms are summarized in Table 2, categorized by their regulatory levels.

**TABLE 2:** Summary of translational and post-translational regulatory mechanisms of GPX4.

Regulatory category	Mechanism subtype	Regulatory factor/event	Core function and mechanism	Reference
Translational regulation	Isoform generation *via* alternative translation initiation	Long isoform (lGPX4)	Contains an N-terminal mitochondrial signal peptide; indispensable for male fertility but cannot rescue embryonic lethality caused by global *Gpx4* knockout.	Liang et al. (2009)
​	​	Short isoform (sGPX4)	Lacks the mitochondrial signal peptide; the core essential isoform for mammalian survival, and also partially localizes to mitochondria to exert antioxidant functions.	Liang et al. (2009)
​	Selenocysteine incorporation and genetic regulation	SECIS element	A stem-loop structure located in the 3′-UTR of *GPX4* mRNA; recruits trans-acting factors to mediate selenocysteine (Sec) insertion at the UGA termination codon during translation.	Stolwijk et al. (2020) and Barayuga et al. (2013)
​	​	SNP rs713041	Located in the 3′-UTR near the SECIS element; alters Sec insertion efficiency and *GPX4* synthesis rate; associated with the risks of multiple cancers, stroke, hypertension, and preeclampsia.	Barbosa et al. (2022) and Peng et al. (2016)
​	​	SNP rs3746162	Located in the 3′-UTR of *GPX4*; a robust independent biomarker for predicting postoperative recurrence in non-muscle invasive bladder cancer (NMIBC).	Ke et al. (2015)
​	​	SNP rs4807542	Forms specific haplotypes with rs713041 that are associated with increased preeclampsia risk; its independent effect remains to be further validated.	Chen et al. (2020) and Liu et al. (2026a)
​	RNA-binding protein-mediated translational regulation	DJ-1	Binds to the 5′-UTR of *GPX4* mRNA to repress its translation under basal conditions; dissociates from *GPX4* mRNA under oxidative stress to rapidly upregulate GPX4 protein synthesis.	Schriever et al. (2017)
​	m^6^A RNA methylation-mediated post-transcriptional regulation	IGF2BP3	An m^6^A reader protein; directly binds to m^6^A-modified *GPX4* mRNA to enhance its stability and translation efficiency, thereby upregulating GPX4 protein levels in glioma.	Deng et al. (2024)
Post-translational regulation	Phosphorylation and autophagy-mediated degradation	Creatine kinase B (CKB)	Functions as a protein kinase to phosphorylate GPX4 at the Ser104 site.	Wu et al. (2023)
​	​	GPX4 Ser104 phosphorylation	Blocks the interaction between GPX4 and HSC70, enabling GPX4 to escape chaperone-mediated autophagy (CMA) degradation and enhancing its protein stability.	Wu et al. (2023)
​	​	Chaperone-mediated autophagy (CMA)	Activated by ferroptosis inducers (e.g., erastin) *via* upregulating LAMP2A; specifically mediates lysosomal degradation of GPX4 to trigger ferroptosis.	Zhu et al. (2025)
​	​	HSP90	A positive regulator of CMA; HSP90 inhibitors stabilize the GPX4 protein and inhibit ferroptosis by blocking CMA activity.	Zhu et al. (2025)
​	Ubiquitination and deubiquitination modifications	LUBAC complex (HOIP)	Catalyzes linear (M1-linked) ubiquitination of GPX4, enhances GPX4 protein stability, and protects it from proteasomal degradation.	Tuo et al. (2026)
​	​	TRIM26	Catalyzes K63-linked non-degradative ubiquitination of GPX4 at Lys107 and Lys117, stabilizes GPX4, and inhibits ferroptosis in glioma cells.	Wang et al. (2023)
​	​	TRIM21	Catalyzes K48-linked degradative ubiquitination of GPX4, mediates GPX4 proteasomal degradation, and promotes ferroptosis in ischemia/reperfusion-induced acute kidney injury.	Sun et al. (2023)
​	​	NEDD4L	Catalyzes K48-linked ubiquitination of GPX4, mediates GPX4 degradation, and confers ferroptosis sensitivity in non-small-cell lung cancer.	Cheng et al. (2023a)
​	​	FBXO31	Catalyzes K48-linked ubiquitination of GPX4, promotes GPX4 proteasomal degradation, and enhances chemosensitivity in cholangiocarcinoma.	Zhu et al. (2022)
​	​	TRIM59	Catalyzes K48-linked ubiquitination of GPX4, mediates GPX4 degradation, and promotes hepatocyte ferroptosis in non-alcoholic fatty liver disease.	Zhang et al. (2023)
​	​	OTUD5	A deubiquitinase that removes K48-linked ubiquitin chains from GPX4, stabilizes GPX4, and inhibits ferroptosis in myocardial and renal ischemia/reperfusion injury.	Liu et al. (2023) and Chu et al. (2023)
​	​	OTUB1	A deubiquitinase recruited by CST1 to GPX4; removes ubiquitin chains from GPX4 to enhance its stability and promote gastric cancer metastasis.	Li et al. (2023)
​	​	USP7	A deubiquitinase that binds and deubiquitinates GPX4; its downregulation mediates macrophage ferroptosis in chronic obstructive pulmonary diseases.	Ruan et al. (2025)
​	​	USP10	Indirectly stabilizes GPX4 *via* upregulating SIRT6, attenuates ferroptosis, and drives malignant progression in thyroid cancer.	Lian et al. (2024)
​	Other post-translational modifications	GPX4 Pro159 hydroxylation	Catalyzed by PHD3 recruited by phosphorylated PSAT1; blocks GPX4-HSC70 interaction to inhibit CMA-mediated GPX4 degradation and confer ferroptosis resistance.	Zheng et al. (2025)

#### 3.2.1 Isoform generation and functional diversification

The translation initiation of GPX4 has some distinctive features as its gene contains two functional ATG start codons that give rise to two major protein isoforms through alternative translation initiation: the long isoform (lGPX4) and the short isoform (sGPX4). lGPX4 is produced from the first ATG codon and includes an N-terminal mitochondrial signal peptide, which has traditionally been thought to direct it to mitochondria. sGPX4, initiated from the second ATG codon, lacks this peptide and was previously considered the predominant non-mitochondrial form found in the cytosol and nucleus (Liang et al., 2009).

*In vivo*, however, the functions and subcellular distribution of these isoforms appear more nuanced than initially appreciated. A key study using transgenic mouse models that express individual isoforms has shed light on their distinct physiological roles. Notably, sGPX4 alone was sufficient to rescue the embryonic lethality associated with complete Gpx4 knockout, establishing it as the essential isoform for survival. Unexpectedly, sGPX4 was also detected in the mitochondria of somatic cells from rescued mice, with a submitochondrial localization pattern similar to that observed in wild-type animals. In contrast, although lGPX4 expression could not rescue embryonic lethality, it proved essential for male fertility (Liang et al., 2009). These observations challenge the traditional view of GPX4 isoform localization and function, suggesting that sGPX4 not only supports organismal viability but also contributes to mitochondrial roles, while lGPX4 appears to serve more specialized functions in certain tissues, such as the testis. In addition, GPX4 has been found in both the cytoplasmic and nuclear compartments of intestinal epithelial cells, with expression levels increasing in both locations during differentiation (Speckmann et al., 2011).

#### 3.2.2 Selenium incorporation and regulatory mechanisms

As a selenoprotein, GPX4 incorporates Sec at its active site through a specialized co-translational mechanism. This process depends on a stem-loop structure within the 3′ untranslated region (UTR) of GPX4 mRNA, known as the selenocysteine insertion sequence (SECIS) element, which recruits specific trans-acting factors and Sec-specific tRNA[Ser]Sec to decode UGA termination codons for Sec incorporation (Stolwijk et al., 2020; Barayuga et al., 2013). Accordingly, cellular selenium availability directly affects GPX4 synthesis efficiency and enzymatic activity. Selenium deficiency restricts GPX4 expression, increasing susceptibility to oxidative stress and ferroptosis, whereas selenium supplementation enhances GPX4 expression and protection against stress-related damage. For example, selenium enrichment increases GPX4 levels and activity in neuronal cells and effectively counteracts methamphetamine-induced oxidative injury (Stolwijk et al., 2020; Barayuga et al., 2013). A recent pan-cancer analysis of approximately 1,000 cell lines identified a correlated functional module comprising GPX4, components of the selenocysteine synthesis pathway, and thioredoxin reductase 1 (TXNRD1), highlighting the importance of the selenoprotein synthesis machinery in maintaining the GPX4–TXNRD1 functional axis (Mao et al., 2021).

A functional SNP near the SECIS element in the GPX4 3′ UTR, rs713041 (GPX4 c.718t), has been shown to affect GPX4 expression by altering SECIS affinity for the Sec incorporation machinery (Barbosa et al., 2022). Selenium supplementation studies indicate that individuals with the TT genotype show smaller increases in lymphocytic GPX1 and plasma GPX3 activities than CC carriers, suggesting reduced selenoprotein synthesis efficiency (Jablonska et al., 2015). This SNP has been associated with various disease risks and prognoses, including breast cancer mortality (Jablonska et al., 2015), bladder cancer subtype susceptibility (Savina et al., 2016), colorectal (Peters et al., 2008) and prostate cancer risks (Abe et al., 2011), as well as stroke and preeclampsia in hypertensive disorders (Peng et al., 2016). A recent meta-analysis confirmed that the T allele is associated with elevated risks for colorectal cancer, stroke, and hypertension, while correlating with a reduced incidence of preeclampsia (Barbosa et al., 2022). Mechanistically, T-allele carriers exhibit pro-inflammatory and pro-adhesive endothelial phenotypes, which may help explain the associations with cardiovascular disease (Polonikov et al., 2012). Notably, the association with preeclampsia appears to be population-specific. In contrast to the meta-analysis findings, a large Chinese Han cohort (1,008 cases and 1,386 controls) identified the C allele as a significant risk factor (OR = 1.216) for preeclampsia, with consistent associations across mild, severe, and early-onset subtypes (Peng et al., 2016). Another Chinese study linked rs713041 variants to preeclampsia susceptibility, with specific haplotypes (GT and GC) formed by rs4807542 increasing disease risk (Chen et al., 2020). Although rs4807542 (t2572c) showed potential three-way interactions with TXN and TXNRD2 in breast cancer, these associations did not remain significant after multiple testing correction (Liu F. et al., 2026). Additionally, rs3746162, another 3′ UTR SNP, has been identified as a potential prognostic biomarker in non-muscle-invasive bladder cancer (NMIBC). In patients treated with transurethral resection alone, variant carriers had a 5.43-fold higher recurrence risk than wild-type individuals, particularly in low-grade tumors. Given its proximity to the SECIS element, this SNP may affect GPX4 mRNA structure, stability, or translational efficiency (Ke et al., 2015).

#### 3.2.3 Post-translational modifications regulating GPX4 stability

Phosphorylation is an important post-translational mechanism that regulates GPX4 stability. Recent studies have uncovered a moonlighting function of creatine kinase B (CKB). Following IGF-1R pathway activation, AKT phosphorylates CKB at T133, which converts it from a metabolic enzyme into a protein kinase that phosphorylates GPX4 at S104. This modification prevents the interaction between GPX4 and HSC70, thereby allowing GPX4 to evade chaperone-mediated autophagy (CMA) degradation and resulting in significantly enhanced GPX4 stability. This ultimately suppresses ferroptosis and promotes tumor growth (Wu et al., 2023). Conversely, CMA activation serves as a direct route for GPX4 degradation and ferroptosis execution. The ferroptosis inducer erastin upregulates lysosome-associated membrane protein 2a (LAMP2A), which activates CMA-mediated GPX4 degradation. This process depends on HSC70-mediated recognition and lysosomal delivery of GPX4. HSP90 functions as a positive regulator of CMA, and its inhibition stabilizes GPX4 levels, thereby protecting cells from ferroptosis (Zhu et al., 2025). Together, these observations illustrate a dual regulatory role for CMA: specific post-translational modifications allow GPX4 to escape CMA degradation, whereas CMA activation directly degrades GPX4 to trigger ferroptosis.

Ubiquitination and deubiquitination are central to the regulation of GPX4 degradation and stability. Multiple E3 ligases and deubiquitinases (DUBs) form a complex regulatory network whose functional outcomes can be broadly categorized by the type of ubiquitin chain linkage and its effect on GPX4 fate. A key stabilizing mechanism involves non-degradative ubiquitination. The linear ubiquitin chain assembly complex (LUBAC) complex, *via* its catalytic subunit HOIP, conjugates linear (M1-linked) linear ubiquitin chain assembly complex to GPX4. Unlike K48-linked chains that typically promote proteasomal degradation, this linear ubiquitination enhances GPX4 stability and protects it from proteasomal breakdown, thereby inhibiting ferroptosis. Accordingly, genetic ablation of LUBAC components reduces GPX4 levels and sensitizes cells to ferroptosis inducers, while loss of the LUBAC-modulating DUB OTULIN produces a similar effect (Tuo et al., 2026). Similarly, the TRIM family E3 ligase TRIM26 catalyzes K63-linked ubiquitination at residues K107 and K117 of GPX4, which also enhances GPX4 stability and suppresses ferroptosis in glioma cells through a non-degradative mechanism (Wang et al., 2023).

In contrast to these stabilizing E3 ligases, a distinct group promotes GPX4 degradation through K48-linked ubiquitination. TRIM21, for instance, is upregulated in ischemia/reperfusion-induced acute kidney injury (AKI) and mediates GPX4 ubiquitination and subsequent proteasomal degradation. Trim21 knockout restores GPX4 levels, reduces ferroptosis and mitochondrial damage in renal tubular cells, and preserves renal function, confirming that TRIM21-mediated GPX4 degradation exacerbates this pathological condition (Sun et al., 2023). This degradative function is shared by NEDD4L, which mediates GPX4 degradation and confers ferroptosis sensitivity in non-small-cell lung cancer. Notably, lactate produced by tumor cells under chemotherapy stress activates the p38–SGK1 axis, which, in turn, suppresses NEDD4L-mediated ubiquitination and degradation of GPX4, thereby contributing to ferroptosis resistance in these cells (Cheng F. et al., 2023). FBXO31 promotes GPX4 proteasomal degradation and enhances chemosensitivity in cholangiocarcinoma (Zhu et al., 2022). TRIM59 mediates GPX4 degradation and promotes hepatocyte ferroptosis in non-alcoholic fatty liver disease (Zhang et al., 2023).

Counterbalancing these E3 ligases, several DUBs maintain GPX4 stability by removing ubiquitin chains. OTUD5 has been identified as a deubiquitinase that protects GPX4 from K48-linked ubiquitination and degradation. However, this protective mechanism is disrupted under stress through distinct routes. In myocardial ischemia/reperfusion injury, 4-HNE accumulation induces carbonylation of both GPX4 and OTUD5, interfering with their interaction and impairing OTUD5-mediated GPX4 deubiquitination (Liu et al., 2023). In renal ischemia/reperfusion injury, mTORC1-mediated autophagy promotes OTUD5 degradation, indirectly destabilizing GPX4 (Chu et al., 2023). OTUB1 represents another DUB that stabilizes GPX4, acting in gastric cancer where CST1 recruits OTUB1 to GPX4, thereby enhancing its stability, reducing ROS accumulation and ferroptosis, and promoting tumor metastasis (Li et al., 2023). In chronic obstructive pulmonary disease (COPD), cigarette smoke-induced DLPC downregulates USP7 expression. Under physiological conditions, USP7 binds GPX4 *via* its TRAF/CAT domain and removes ubiquitin chains to maintain GPX4 stability. Downregulation of USP7 leads to increased GPX4 ubiquitination and degradation, which, in turn, promotes ferroptosis in macrophages (Ruan et al., 2025). In thyroid cancer, USP10 has been reported to enhance GPX4 stability by upregulating SIRT6 expression, thereby attenuating ferroptosis and contributing to tumor progression (Lian et al., 2024).

Other modifications also support GPX4 stability. Hydroxylation has recently emerged as a modification that supports GPX4 stability. In the tumor microenvironment, interferon-γ (IFNγ) activates Ca^2+^/calmodulin-dependent protein kinase II (CAMKII), which then phosphorylates the serine aminotransferase PSAT1. Phosphorylated PSAT1 associates with GPX4 and recruits the prolyl hydroxylase PHD3 to hydroxylate GPX4 at proline 159 (P159). This hydroxylation interferes with the interaction between HSC70 and GPX4, thereby suppressing autophagy-mediated degradation of GPX4, enhancing its stability, and contributing to ferroptosis resistance (Zheng et al., 2025). Protein carbonylation represents an irreversible form of oxidative damage induced by ROS. Although loss of GPX4 activity results in lipid peroxide accumulation and subsequent carbonylation of other proteins, such as STING (Chen Y. et al., 2025), whether GPX4 itself undergoes carbonylation and whether this modification would affect its function are open questions that require direct experimental testing.

#### 3.2.4 Other modifications and regulatory mechanisms

In the tumor microenvironment, IFNγ activates Ca^2+^/calmodulin-dependent protein kinase II (CAMKII), which then phosphorylates the serine aminotransferase PSAT1. Phosphorylated PSAT1 associates with GPX4 and recruits the prolyl hydroxylase PHD3 to hydroxylate GPX4 at proline 159 (P159). This hydroxylation interferes with the interaction between HSC70 and GPX4, thereby suppressing autophagy-mediated degradation of GPX4, enhancing its stability, and contributing to ferroptosis resistance (Zheng et al., 2025). Protein carbonylation represents an irreversible form of oxidative damage induced by ROS. Although loss of GPX4 activity results in lipid peroxide accumulation and subsequent carbonylation of other proteins, such as STING (Chen Y. et al., 2025), whether GPX4 itself undergoes carbonylation and whether such modification would affect its function are open questions that require direct experimental testing.

#### 3.2.5 Translational regulation mediated by RNA-binding proteins

In addition to the modifications discussed above, GPX4 translation is dynamically regulated by RNA-binding proteins, offering another layer of control that may facilitate rapid cellular responses under stress. The Parkinson’s disease (PD)-associated protein DJ-1 serves as an example in this context. DJ-1 binds specifically to GG/CC-rich sequence motifs in the 5′ untranslated region (5′-UTR) of GPX4 mRNA. Under basal conditions, this binding mildly suppresses GPX4 translation, as evidenced by increased GPX4 protein levels in DJ-1 knockdown cells. Upon oxidative stress, DJ-1 undergoes oxidative modification and dissociates from GPX4 mRNA. This release alleviates translational repression and allows rapid GPX4 synthesis, thereby enhancing the cellular capacity to counter lipid peroxidation damage. Notably, pathogenic DJ-1 mutants linked to Parkinson’s disease show impaired RNA binding. This regulatory axis thus reveals a translational control mechanism for GPX4 and offers a potential molecular link to the neuroprotective functions of DJ-1 and its broader role in oxidative stress responses (Schriever et al., 2017).

#### 3.2.6 Regulation mediated by m6A RNA methylation

**FIGURE 2:**
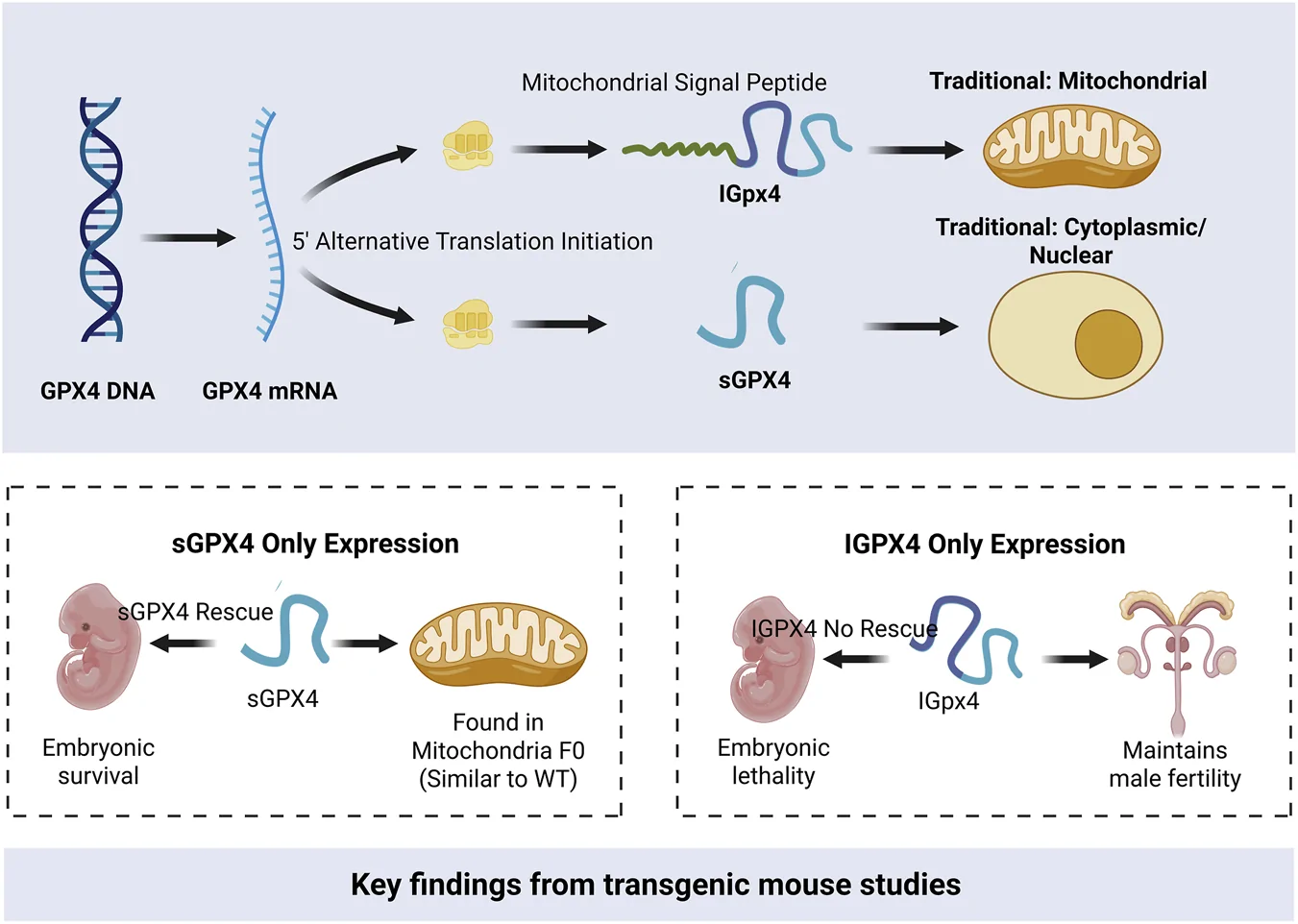
Generation and functional differentiation of GPX4 isoforms via alternative translation initiation. The 5’ alternative translation initiation of GPX4 mRNA produces two distinct isoforms: the long isoform lGPX4 with an N‐terminal mitochondrial signal peptide (translated from the first ATG), and the short isoform sGPX4 without the signal peptide (translated from the second ATG). Transgenic mouse studies demonstrate that sGPX4 is essential for embryonic survival and can partially localize to mitochondria, whereas lGPX4 is dispensable for embryonic development but exerts a non-redundant role in maintaining male fertility. This isoform‐specific functional specialization underlies the tissue-specific regulatory capacity of GPX4 in physiological homeostasis and ferroptosis‐related pathology.

N6-methyladenosine (m6A) is the most abundant internal modification on mRNA and represents another important post-transcriptional layer of gene regulation. This modification is dynamically controlled by writer proteins (methyltransferases), eraser proteins (demethylases), and reader proteins that recognize m6A sites and mediate downstream effects. In glioma, the m6A reader IGF2BP3 has been identified as a regulator of GPX4 expression and ferroptosis. IGF2BP3 is highly expressed in glioma and correlates with poor patient prognosis. Mechanistically, IGF2BP3 binds directly to an m6A motif on GPX4 mRNA and enhances both its stability and translation efficiency. This results in elevated GPX4 protein levels, which protect glioma cells from ferroptosis and support their growth and survival. Knockdown of IGF2BP3 reduces the GPX4 protein, promotes lipid peroxide accumulation, and induces ferroptosis, and this phenotype can be reversed by restoring GPX4 expression. Clinical sample analyses show a positive correlation between IGF2BP3 and GPX4 protein levels in glioma tissues, but not between their mRNA levels, which further supports a post-transcriptional mechanism. These findings point to a route through which m6A modification regulates GPX4 *via* IGF2BP3 and may offer new perspectives for therapeutic strategies in glioma (Deng et al., 2024) [Figure 2].

The 5′ alternative translation initiation of GPX4 mRNA produces two distinct isoforms: the long isoform lGPX4 with an N-terminal mitochondrial signal peptide (translated from the first ATG) and the short isoform sGPX4 without the signal peptide (translated from the second ATG). Transgenic mouse studies demonstrate that sGPX4 is essential for embryonic survival and can partially localize to mitochondria, whereas lGPX4 is dispensable for embryonic development but exerts a non-redundant role in maintaining male fertility. This isoform-specific functional specialization underlies the tissue-specific regulatory capacity of GPX4 in physiological homeostasis and ferroptosis-related pathology.

#### 3.2.7 RNA-mediated post-transcriptional regulation

Beyond translation initiation and mRNA stability regulated by RNA-binding proteins, GPX4 expression is also modulated by multiple RNA-mediated mechanisms, including non-coding RNAs and m^6^A methylation.

MicroRNAs (miRNAs) directly repress GPX4 translation by targeting its 3′ UTR. miR-324-3p has been validated to directly bind GPX4 mRNA and suppress its expression, and this axis mediates metformin-induced ferroptosis in breast cancer cells (Hou et al., 2021). miR-1231 and miR-6805 also directly target GPX4 transcripts, with circKIF4A and circ0060467 functioning as competing endogenous RNAs (ceRNAs) to sequester these miRNAs and thereby relieve GPX4 repression in thyroid cancer and hepatocellular carcinoma, respectively (Tan et al., 2024; Chen W. et al., 2021). This ceRNA network illustrates a layered regulatory logic in which circular RNAs indirectly upregulate GPX4 by depleting repressive miRNAs.

In glioma, the m^6^A reader IGF2BP3 directly binds to m^6^A-modified GPX4 mRNA and enhances its stability and translational efficiency, leading to elevated GPX4 protein levels without changing mRNA abundance. Knockdown of IGF2BP3 reduces the GPX4 protein, promotes lipid peroxide accumulation, and induces ferroptosis, and this effect is reversed by GPX4 restoration (Deng et al., 2024). This m^6^A-dependent mechanism represents a post-transcriptional regulatory layer that operates independently of miRNA-mediated repression.

Long non-coding RNAs have also been implicated in GPX4 regulation, although the mechanistic evidence is less extensive. In the context of ferroptosis and the GSH–GPX4 axis, a systematic review has mapped an extensive non-coding RNA network that includes multiple lncRNAs acting as ceRNAs or transcriptional modulators (Hussain et al., 2024). In addition, an internal ribosome entry site (IRES) in the GPX4 5′ UTR sustains GPX4 synthesis under oxidative stress and nutrient deprivation, when cap-dependent translation is globally suppressed (Zhong et al., 2025).

Collectively, these RNA-mediated mechanisms—miRNA-directed repression, circRNA-mediated miRNA sponging, m^6^A-dependent mRNA stabilization, and IRES-driven translation—constitute an integrated post-transcriptional regulatory layer that modulates GPX4 abundance and ferroptosis sensitivity across diverse cellular contexts. Notably, these mechanisms are not mutually exclusive; in certain tumors, multiple layers (e.g., circRNA sponging and m^6^A stabilization) may co-exist and jointly determine GPX4 protein levels.

### 3.3 Regulation of GPX4 by metabolism and small molecules

The activity and stability of GPX4 are also influenced by various endogenous metabolites, including essential trace elements, and by exogenous small molecules. Copper ions, for instance, appear to act as a dual regulator: physiological levels support normal cellular function, whereas pathological overload resulting from dysregulated homeostasis or external exposure can compromise GPX4 stability. In the context of copper overload, copper ions have been shown to bind directly to cysteine residues C107 and C148 of GPX4, promote its aggregation and ubiquitination, and lead to its degradation through the TAX1BP1-mediated autophagic pathway. This mechanism is distinct from the canonical cuproptosis pathway and points to a specific role for copper in GPX4 regulation (Xue et al., 2023).

Lactate produced by tumor cells under chemotherapy stress activates the p38–SGK1 axis, which, in turn, suppresses NEDD4L-mediated ubiquitination and degradation of GPX4, thereby contributing to ferroptosis resistance in these cells (Cheng F. et al., 2023).

A range of small-molecule compounds have been developed to target GPX4. RSL3 is among the most widely studied GPX4 inhibitors; it covalently binds to the selenocysteine residue in the active site, irreversibly inhibiting GPX4 activity, and has become a standard tool for inducing ferroptosis. Some endoperoxide compounds, such as FINO_2_, act through a dual mechanism: they directly inactivate GPX4 while also oxidizing ferrous iron (Fe^2+^), which together amplifies lipid peroxidation (Gaschler et al., 2018). These small molecules may offer useful strategies for targeting GPX4 in diseases, particularly in malignancies.

**FIGURE 3:**
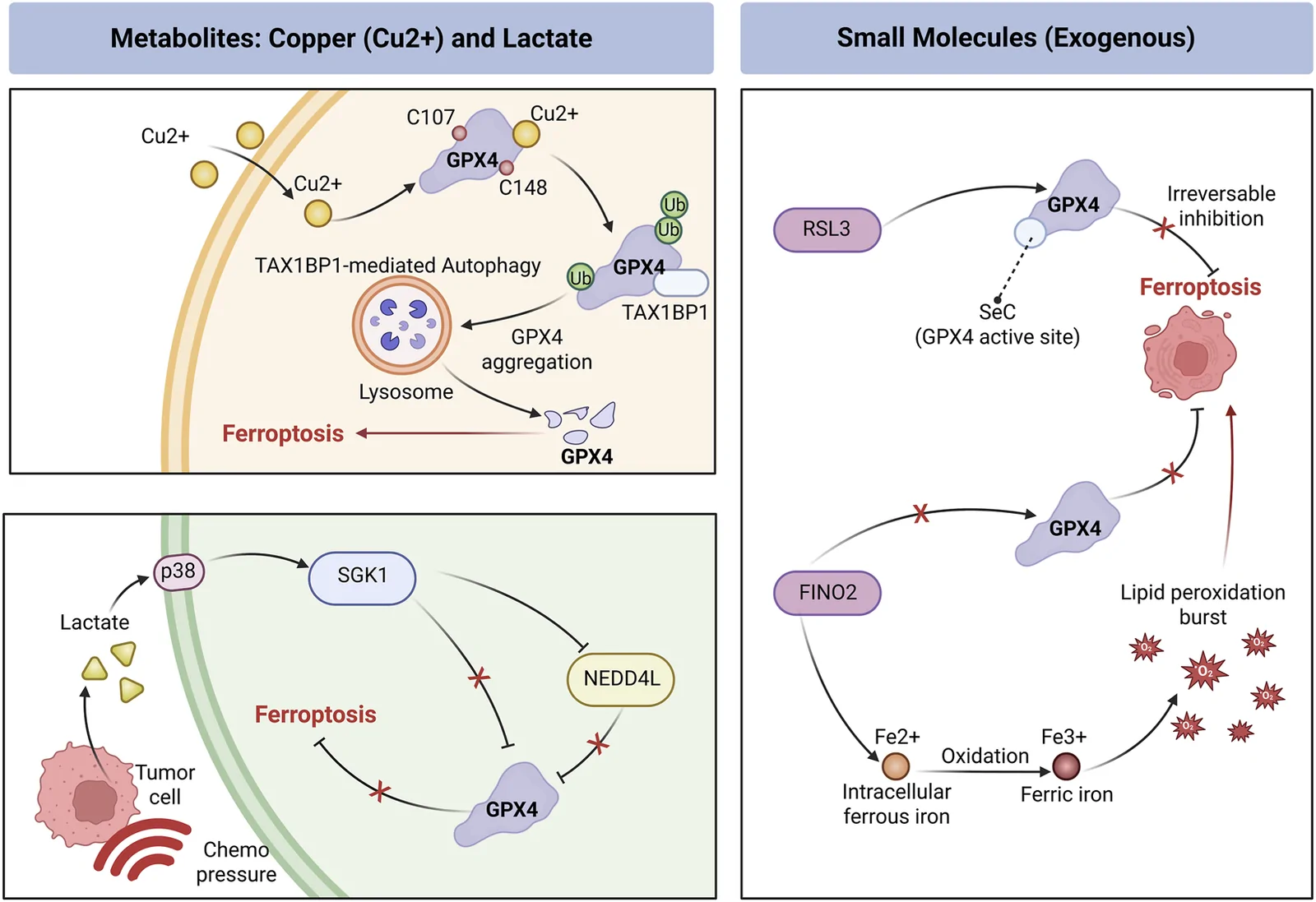
Regulation of GPX4 by endogenous metabolites and exogenous small molecules in ferroptosis. Endogenous copper (Cu^2+^) at pathological overload concentrations promotes GPX4 ubiquitination and TAX1BP1‐mediated autophagic degradation to facilitate ferroptosis, while lactate stabilizes GPX4 via the p38‐SGK1‐NEDD4L axis to inhibit ferroptosis. Representative exogenous small molecules, including the irreversible GPX4 covalent inhibitor RSL3 and the dual-acting GPX4 inhibitor FINO2, directly inactivate GPX4 and trigger robust ferroptosis. This landscape summarizes both the metabolic regulatory network of GPX4 and the core chemical strategies for GPX4 targeting in ferroptosis modulation.

In contrast to these inhibitors, certain natural small molecules have been reported to exert protective effects by upregulating GPX4 expression. For example, in a cellular model of diabetic nephropathy, platycodin D, a triterpenoid saponin, was found to counteract high-glucose-induced ferroptosis in renal tubular epithelial cells. This effect appears to be mediated by reversing the high-glucose-induced downregulation of GPX4 mRNA and protein, thereby restoring the cellular capacity to counteract lipid peroxidation (Lin et al., 2023) (Figure 3).

Endogenous copper (Cu^2+^) at pathological overload concentrations promotes GPX4 ubiquitination and TAX1BP1-mediated autophagic degradation to facilitate ferroptosis, while lactate stabilizes GPX4 *via* the p38–SGK1–NEDD4L axis to inhibit ferroptosis. Representative exogenous small molecules, including the irreversible GPX4 covalent inhibitor RSL3 and the dual-acting GPX4 inhibitor FINO2, directly inactivate GPX4 and trigger robust ferroptosis. This landscape summarizes both the metabolic regulatory network of GPX4 and the core chemical strategies for GPX4 targeting in ferroptosis modulation.

## 4 Protein–protein interaction network of GPX4

A fuller understanding of GPX4 function in complex biological contexts depends not only on its enzymatic properties but also on knowledge of its interacting partners. Proteomic studies have identified proteins that associate with GPX4, which provide information about its regulation and its possible involvement in broader cellular processes. These interacting proteins and their reported functions are listed in Table 3.

**TABLE 3:** Summary of key GPX4-interacting and functionally related proteins.

Protein name	Description/function	Reference
Peroxiredoxin 6 (PRDX6)	Peroxiredoxin 6 positively correlated with the “core selenium functional module” where GPX4 resides and may synergistically regulate lipid peroxidation.	Mao et al. (2021)
Stearoyl-CoA desaturase (SCD)	Stearoyl-CoA desaturase exhibits a significant negative correlation (antagonistic effect) with the GPX4 module and prevents lipid peroxidation at the source by generating monounsaturated fatty acids.	Mao et al. (2021)
Fatty acid synthase (FASN)	Fatty acid synthase has a positive genetic interaction with SCD, and its deficiency reduces cellular dependence on SCD.	Du et al. (2025)
TRIM59	E3 ubiquitin ligase; enhances the ubiquitination and degradation of GPX4 to promote ferroptosis in hepatocytes in non-alcoholic fatty liver disease.	Zhang et al. (2023)
TRIM21	E3 ubiquitin ligase; mediates the ubiquitination and degradation of GPX4 to exacerbate ischemia/reperfusion injury in acute kidney injury.	Sun et al. (2023)
FBXO31	E3 ubiquitin ligase; enhances cellular sensitivity to cisplatin by promoting proteasomal degradation of GPX4 in cholangiocarcinoma.	Zhu et al. (2022)
HOIP	Core component of the LUBAC complex; enhances GPX4 protein stability and inhibits ferroptosis by mediating linear ubiquitination of GPX4.	Tuo et al. (2026)
TRIM26	E3 ubiquitin ligase; enhances GPX4 protein stability and inhibits ferroptosis in glioma cells by catalyzing K63-linked non-degradative ubiquitination of GPX4.	Wang et al. (2023)
TAK1 complex	Interacts with GPX4 to modulate TAK1 kinase activity, activates the downstream MAPK/JNK and NF-κB signaling pathways, and participates in the progression of EBV-associated nasopharyngeal carcinoma.	Yuan et al. (2022)
FUNDC1	Mitophagy receptor; directly binds to GPX4 and mediates its mitochondrial transport and autophagic degradation, thereby regulating hepatic ferroptosis and fibrosis.	Bi et al. (2024)
Glutathione S-transferase Mu 1 (GSTM1)	Forms a complex with GPX4 to drive platinum-based chemoresistance in lung cancer brain metastases.	Liu et al. (2021a)
Glutathione S-transferase Mu 3 (GSTM3)	Directly binds to and inhibits GPX4, promotes radiotherapy-induced ferroptosis in nasopharyngeal carcinoma cells, and enhances radiosensitivity.	Chen et al. (2024a)

### 4.1 Systematic identification of GPX4-interacting proteins

The construction of the GPX4 interaction network has been greatly facilitated by advances in proteomic technologies, and a range of high-throughput approaches have now been applied to systematically identify proteins associated with GPX4.

Affinity purification coupled with mass spectrometry (AP–MS), including conventional co-immunoprecipitation followed by mass spectrometry (Co–IP/MS), remains a core strategy for characterizing stable protein complexes. In this approach, specific antibodies or affinity tags are used to enrich GPX4 and its associated partners, which are subsequently identified by sensitive mass spectrometry. To mitigate the issue of nonspecific background binding, several computational tools have been developed, such as CompPASS, CRAPOME, and the SAINT scoring algorithm. These platforms apply unbiased comparative scoring to filter out noise and enable the selection of high-confidence candidate interactors (Sowa et al., 2009). The establishment of these methods has supported large-scale, proteome-wide protein–protein interaction projects such as the BioPlex network, and has also provided useful resources for the specific screening of GPX4-binding proteins (Huttlin et al., 2017; Huttlin et al., 2015).

As a complementary approach to AP–MS, high-throughput yeast two-hybrid (Y2H) screening, particularly array-based recombinant Y2H, offers a powerful means to detect direct, binary protein–protein interactions. Because the assay takes place in the yeast nucleus, it can effectively exclude indirect associations that are mediated by RNA molecules, thus allowing more accurate identification of direct binding between GPX4 and its target proteins (Lundrigan et al., 2026).

For the detection of weak, transient, or spatially proximal interactions, proximity-dependent biotinylation techniques have been developed. In these methods, a biotin ligase is fused to GPX4 and catalyzes the biotinylation of proteins within approximately 10–15 nm of the active site. The labeled proteins can then be affinity-purified and identified by mass spectrometry, allowing the construction of a proximity interaction landscape for GPX4. Such approaches have been successfully used to map protein topological networks in organelles and even entire cells (Go et al., 2021; Chu et al., 2024). More recently, emerging strategies such as photoactivation-dependent proximity labeling (PDPL) have provided the means to analyze protein interaction networks with improved spatiotemporal resolution (Liu L. et al., 2024).

Other complementary methods also contribute to this effort. Co-fractionation coupled with mass spectrometry (CF/MS) enables the identification of protein complexes under native conditions and can reveal potential components of GPX4-containing complexes by analyzing the correlation of protein elution profiles across biochemical fractions (Zeng et al., 2025). In addition, genetic interaction maps derived from genome-wide CRISPR screening and co-essentiality analyses can uncover proteins and pathways that function synergistically or antagonistically with GPX4 at the functional level. These approaches allow the construction of a functional interaction network for GPX4 without requiring direct physical binding (Mao et al., 2021).

### 4.2 Key interaction partners and their functions

Based on the protein–protein interactions identified through the techniques described above, the GPX4-interacting partners can be broadly grouped into three functional categories: those that modulate GPX4 stability, those involved in signal transduction, and those that cooperate with GPX4 to execute specific physiological functions.

Regarding functionally synergistic and antagonistic interacting proteins, a large-scale co-essentiality analysis has systematically delineated a “core selenium functional module” centered on GPX4. This study, conducted across nearly one thousand cancer cell lines, revealed that GPX4 is functionally tightly coupled not only with all seven known components of the selenocysteine biosynthesis and insertion machinery but also with the key oxidoreductase TXNRD1. The similar effects on cell viability observed upon genetic disruption of these genes suggest that, in these cancer cells, a primary role of the entire selenoprotein synthesis system is to support the expression of GPX4 and TXNRD1. In addition, the same analysis identified two previously uncharacterized functionally linked proteins: peroxiredoxin 6 (PRDX6), which shows a positive correlation with this module, and stearoyl-CoA desaturase (SCD), which exhibits a significant negative correlation with the module, particularly with GPX4. These correlations indicate the potential synergistic or antagonistic roles of these proteins in the regulation of lipid peroxidation and ferroptosis (Mao et al., 2021). Such a functional antagonism is consistent with their parallel yet opposing actions in counteracting lipid peroxidation: GPX4 detoxifies lipid peroxides, whereas SCD acts upstream by generating monounsaturated fatty acids, which are less prone to peroxidation. However, systematic genetic interaction maps have offered a more nuanced view of their interconnections within the broader metabolic network. For instance, a positive genetic interaction has been observed between the fatty acid synthase (FASN) and SCD, indicating that FASN-deficient cells show reduced dependency on SCD possibly because decreased synthesis of saturated fatty acids, the substrates of SCD, mitigates the deleterious effects of SCD deficiency (Du et al., 2025). Taken together, these large-scale genetic analyses have uncovered a complex balance among fatty acid synthesis, desaturation, and peroxide detoxification under conditions of metabolic stress.

With respect to proteins that regulate GPX4 stability, this group forms a central part of the GPX4 interaction network and includes E3 ubiquitin ligases, deubiquitinases, kinases, molecular chaperones, and autophagy-related proteins. E3 ubiquitin ligases and deubiquitinases directly influence the fate of GPX4 through ubiquitination. For example, HOIP and TRIM26 enhance GPX4 stability *via* non-degradative ubiquitination, whereas TRIM21 and FBXO31 promote its proteasomal degradation through degradative ubiquitination (Tuo et al., 2026; Wang et al., 2023; Sun et al., 2023; Zhu et al., 2022). The kinase CKB blocks the interaction between GPX4 and HSC70 through phosphorylation, thereby inhibiting CMA-dependent degradation (Wu et al., 2023). The chaperones HSC70 and HSP90 regulate the efficiency of lysosomal degradation of GPX4 by modulating CMA activity (Zhu et al., 2025). In addition, the autophagy receptor TAX1BP1 can specifically recognize aggregated GPX4 and mediate its autophagic-lysosomal degradation (Xue et al., 2023).

Regarding interacting proteins involved in signal transduction, GPX4 not only functions as an executor of ferroptosis but also participates in key signaling pathways through protein–protein interactions. In Epstein–Barr virus-positive nasopharyngeal carcinoma, GPX4 directly associates with the TAK1 kinase complex, which includes TAK1, TAB1, and TAB3, and modulates TAK1 activity. This interaction, in turn, activates downstream MAPK/JNK and NF-κB signaling, thereby linking cellular redox status to inflammatory and tumor progression pathways (Yuan et al., 2022).

**FIGURE 4:**
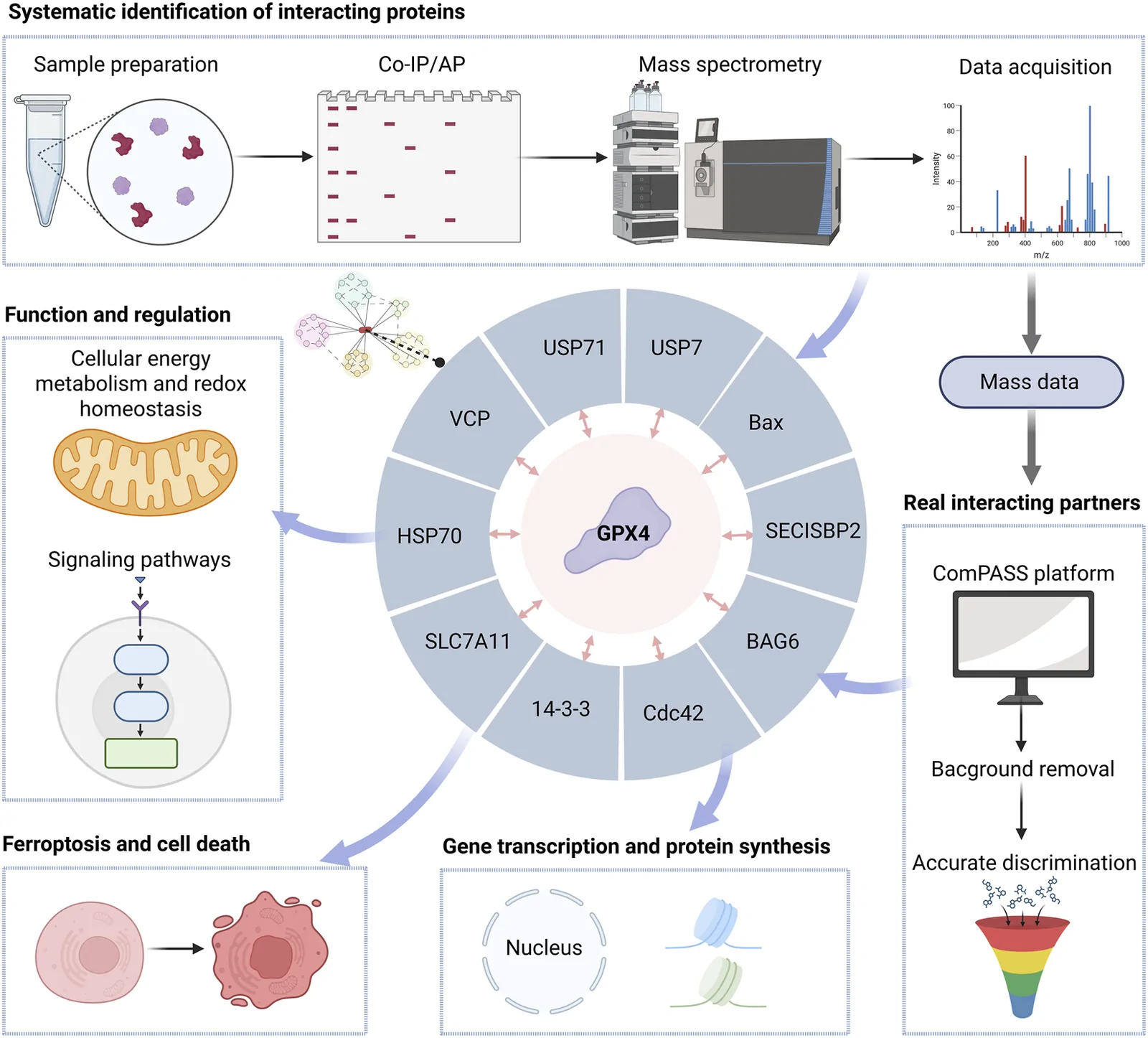
Systematic screening and functional annotation of the GPX4 protein‐protein interaction (PPI) network. A GPX4‐centered PPI network is constructed with representative functional interactors displayed, and the core biological processes regulated by these PPIs are annotated, including cellular redox homeostasis, signal transduction, ferroptosis and cell death, and gene expression regulation. This network provides a comprehensive landscape of GPX4‐mediated functional regulatory networks in physiological and pathological contexts.

Regarding proteins that synergistically execute specific functions, GPX4 can form functional complexes with various partners to coordinate particular biological outcomes. The mitophagy receptor FUNDC1 directly binds to cytoplasmic GPX4 through its amino acid residues 96–133, promotes its mitochondrial import, and mediates its degradation *via* mitophagy, thereby regulating hepatocyte ferroptosis and liver fibrosis progression (Bi et al., 2024). In lung cancer brain metastasis, GPX4 associates with glutathione S-transferase Mu 1 (GSTM1) to drive acquired platinum-based chemoresistance (Liu W. et al., 2021). In nasopharyngeal carcinoma, glutathione S-transferase Mu 3 (GSTM3) directly interacts with GPX4 and inhibits its activity, thus promoting radiotherapy-induced ferroptosis and enhancing tumor radiosensitivity (Chen Y. et al., 2024) (Figure 4).

A GPX4-centered PPI network is constructed with representative functional interactors displayed, and the core biological processes regulated by these PPIs are annotated, including cellular redox homeostasis, signal transduction, ferroptosis and cell death, and gene expression regulation. This network provides a comprehensive landscape of GPX4-mediated functional regulatory networks in physiological and pathological contexts.

## 5 Core roles of GPX4 in physiological and pathological processes

The unique biological functions of GPX4 are rooted in its distinctive molecular structure and substrate specificity. Unlike other members of the glutathione peroxidase family, which predominantly act on soluble peroxides, GPX4 is capable of accessing and reducing lipid hydroperoxides embedded within biological membranes, a property that accounts for its central role in the regulation of ferroptosis. The physiological functions of GPX4 extend to several key processes, including embryonic development, reproduction, and the maintenance of immune homeostasis. Conversely, its dysfunction is closely linked to the pathogenesis and progression of a broad range of major human diseases, such as cancer, neurodegenerative disorders, autoimmune diseases, and organ injury. A summary of the core functions of GPX4 in various physiological and pathological contexts is provided in Table 4.

**TABLE 4:** Summary of the core functions of GPX4 in distinct physiological and pathological processes.

Context/background	Description/function	Reference
Comparison of GPX isoform functions	GPX4 is the only isoform that efficiently reduces phospholipid hydroperoxides; GPX1 exhibits higher catalytic efficiency for small soluble hydroperoxides (e.g., H_2_O_2_).	Schwarz et al. (2023)
Sperm maturation and male fertility	Exerts dual roles: acts as an antioxidant enzyme to protect sperm membranes in early stages; transforms into a key structural protein of the mitochondrial capsule after maturation; the long isoform (lGpx4) is essential for normal sperm morphology and male fertility.	Liang et al. (2009), Ursini et al. (1999), and Foresta et al. (2002)
Sperm maturation and male fertility (clinical correlation)	Reduced GPX4 levels in sperm from infertile men, which correlates with sperm morphology and embryo quality; HBsAg induces ferroptosis in Sertoli cells *via* downregulating GPX4, thereby impairing fertility.	Foresta et al. (2002), Garrido et al. (2004), Meseguer et al. (2006), and Pan et al. (2024)
Immune system homeostasis (systemic lupus erythematosus)	Acts as a protective factor by inhibiting ferroptosis in neutrophils, thereby preventing the onset and progression of systemic lupus erythematosus (SLE).	Li et al. (2021a)
Immune system homeostasis (iNKT cells)	Maintains the survival of invariant natural killer T (iNKT) cells, particularly the IFN-γ-producing NKT1 subset, and protects them from ferroptosis.	Sok et al. (2024)
Immune system homeostasis (anti-tumor CD8^+^ T cells)	Essential for the survival and effector function of anti-tumor CD8^+^ T cells and is a prerequisite for their anti-tumor efficacy.	Chen et al. (2024b)
Cell differentiation and development (embryonic development)	Essential for embryonic development; whole-body knockout results in mid-gestational embryonic lethality. The short isoform (sGpx4) is critical for organismal survival.	Liang et al. (2009)
Cell differentiation and development (human genetic disease)	Loss-of-function mutations in *GPX4* cause a lethal neonatal disorder, Sedaghatian-type spondylometaphyseal dysplasia (SSMD).	Smith et al. (2014)
Cell differentiation and development (intestinal epithelial cells)	Expression is upregulated during intestinal epithelial cell differentiation, enhancing cellular defense against oxidative stress.	Speckmann et al. (2011)
Cell differentiation and development (erythroid lineage)	Promotes enucleation of erythroid progenitor cells *via* regulating lipid raft organization in a ferroptosis-independent manner; maintains the integrity of the mature erythrocyte membrane.	Ouled-Haddou et al. (2020) and Stolwijk et al. (2021)
Platelet function regulation	Cooperates with GPX1 to regulate the arachidonate 12-lipoxygenase metabolic pathway, directs the flux of platelet lipid metabolism by controlling hydroperoxide tone, and modulates thrombosis.	Sutherland et al. (2001)

### 5.1 Physiological functions

#### 5.1.1 Sperm maturation and male fertility

GPX4 serves two distinct and non-redundant functions in the male reproductive system, acting both as an antioxidant enzyme and as a structural protein, and is essential for spermatogenesis, sperm maturation, and fertilization capacity. During early spermatid development, GPX4 exists as a soluble enzyme and exerts its canonical peroxidase activity to protect sperm cell membranes from lipid peroxidation damage (Ursini et al., 1999). During sperm maturation, GPX4 gradually loses its enzymatic activity and undergoes oxidative cross-linking, transitioning into an insoluble structural protein that becomes a major component of the mitochondrial capsule in the sperm midpiece, accounting for more than 50% of the total protein in this structure (Ursini et al., 1999; Foresta et al., 2002). This structural transition appears to be important for maintaining the mechanical stability of the mitochondrial capsule, as well as the normal morphology and motility of sperm.

Studies using transgenic mouse models with isoform-specific expression have helped delineate the functional division between the long and short GPX4 isoforms. The long isoform (lGpx4), which is predominantly expressed in the testis, is indispensable for normal sperm morphology and male fertility, whereas male Gpx4-null mice that express only the short isoform (sGpx4) are infertile (Liang et al., 2009). Insufficient GPX4 levels resulting from selenium deficiency directly induce morphological alterations and impaired motility of the sperm midpiece, leading to male infertility (Ursini et al., 1999; Foresta et al., 2002).

Clinical studies have further supported the association between GPX4 and male fertility in humans. Sperm GPX4 activity and protein content are significantly lower in infertile men than in normozoospermic fertile men, and its levels correlate positively with the proportion of morphologically normal sperm (Foresta et al., 2002; Garrido et al., 2004). In assisted reproductive technology, sperm GPX4 activity has been found to correlate with subsequent embryo quality (Meseguer et al., 2006). Proteomic studies have identified GPX4 as one of the N-linked glycosylated proteins in the human sperm head, suggesting a possible direct involvement in sperm–egg recognition and binding (Wang et al., 2013). In addition, polymorphisms in the GPX4 gene have been reported to be associated with the risk of male infertility (Maiorino et al., 2003).

A range of exogenous factors, including environmental toxicants, clinical medications, and unhealthy lifestyle habits, can also impair male fertility by targeting GPX4. Hepatitis B virus surface antigen (HBsAg) induces ferroptosis in testicular Sertoli cells through downregulation of GPX4, representing one mechanism underlying fertility impairment in male HBV carriers (Pan et al., 2024). Heavy metals, endocrine-disrupting chemicals, platinum-based chemotherapeutics, and cigarette smoke have all been shown to disrupt testicular GPX4 homeostasis through transcriptional repression, ubiquitin-mediated degradation, or glutathione cofactor depletion, leading to impaired spermatogenesis and reduced sperm quality (Su et al., 2024; Ruan et al., 2025; Xue et al., 2023; Wu X. et al., 2022; Kim et al., 2020).

#### 5.1.2 Maintenance of immune system homeostasis

GPX4-mediated regulation of ferroptosis contributes to the homeostasis of both innate and adaptive immunity and appears to influence the survival and functional performance of several immune cell subsets.

In the context of innate immunity, GPX4 serves as a protective factor for neutrophil survival. In SLE, transcriptional repression of GPX4 triggers ferroptosis in neutrophils, which leads to the release of neutrophil extracellular traps (NETs) and contributes to the initiation and progression of autoimmune responses (Li et al., 2021a). In addition, GPX4 regulates the activation of the cGAS–STING innate immune pathway by maintaining cellular redox homeostasis and has been identified as a regulatory node in host antiviral immune responses against DNA viruses (Chen Y. et al., 2025).

In adaptive immunity, GPX4 is required for the homeostasis and function of T cell subsets. It supports the survival of invariant natural killer T (iNKT) cells, particularly the IFN-γ-producing NKT1 subset; deletion of GPX4 results in ferroptosis of iNKT cells and impairs their homeostasis, as well as their anti-tumor and anti-infective functions *in vivo* (Sok et al., 2024). For anti-tumor CD8^+^ effector T cells, GPX4 appears to be a prerequisite for their survival and effector functions. Adenosine A2A receptor signaling has been shown to induce ferroptosis in CD8^+^ T cells by suppressing the glutathione/GPX4 axis, representing a potential mechanism of tumor immunosuppression (Chen S. et al., 2024).

#### 5.1.3 Cell differentiation and embryonic development

GPX4 is required for mammalian embryonic development. Whole-body Gpx4 knockout mice die at mid-gestation, around embryonic day 7.5, and fail to complete normal development (Liang et al., 2009). Transgenic expression of the short isoform sGpx4 fully rescues this embryonic lethality, indicating that sGpx4 is the core isoform supporting embryonic development and organismal survival and that mitochondrial GPX4 function is essential for mammalian viability (Liang et al., 2009).

In humans, loss-of-function mutations in GPX4 cause Sedaghatian-type spondylometaphyseal dysplasia (SSMD), a lethal neonatal genetic disorder characterized by skeletal abnormalities and multi-organ failure. This further supports the importance of GPX4 in human embryonic development and organogenesis (Smith et al., 2014).

GPX4 expression also appears to correlate with cell differentiation. During intestinal epithelial cell differentiation, GPX4 mRNA and protein levels increase significantly as cells mature, a process driven by transcriptional activation through a composite CREB/C/EBPα binding site. The higher GPX4 levels observed in terminally differentiated intestinal epithelial cells may confer enhanced protection against oxidative stress (Speckmann et al., 2011). In the erythroid lineage, GPX4 promotes enucleation of erythroid progenitor cells by regulating lipid raft organization and aggregation at the cleavage furrow, and this function appears to be independent of ferroptosis (Ouled-Haddou et al., 2020). In addition, GPX4 contributes to the maintenance of membrane integrity in mature erythrocytes, and its abundance has been found to correlate negatively with the hemolysis rate of erythrocytes during blood bank storage (Stolwijk et al., 2021).

#### 5.1.4 Regulation of arachidonic acid metabolism and platelet function

GPX4 appears to function as a molecular switch in the biosynthesis of certain signaling lipids, with a particularly notable role in the arachidonic acid metabolic pathway in platelets. One study first confirmed that the GPX4 protein is stably expressed and enzymatically active in mature platelets, even though its mRNA is only detectable in megakaryocytes, the precursor cells of platelets. This observation suggests that the GPX4 protein has a long half-life and sustained function in anucleate platelets (Sutherland et al., 2001).

The main finding of that study is that GPX4, together with cytosolic GPX1, co-regulates a bifurcation point in the 12-lipoxygenase (12-LOX) metabolic pathway. Under normal physiological conditions, these two peroxidases efficiently reduce the intermediate product 12-hydroperoxyeicosatetraenoic acid (12-HpETE), generated by 12-LOX catalysis, to the more stable 12-hydroxyeicosatetraenoic acid (12-HETE), thereby maintaining a low intracellular “hydroperoxide tone.” When GPX activity is inhibited, however, 12-HpETE accumulates substantially, and this accumulation drives a shift in the 12-LOX pathway from the reductive route toward the isomerization route, which converts 12-HpETE into bioactive signaling molecules known as hepoxilins.

**FIGURE 5:**
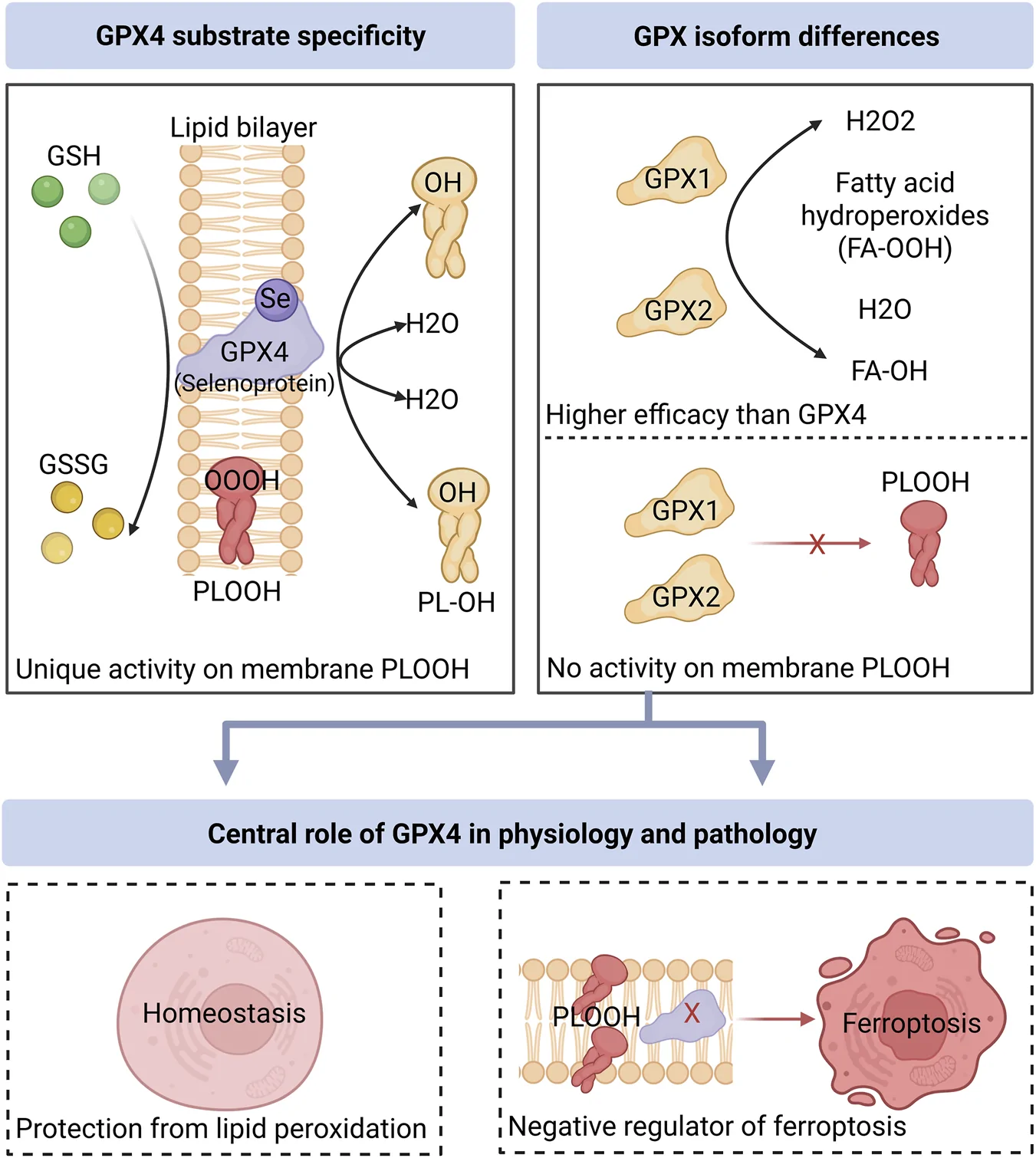
Enzymatic properties of GPX4 and its core roles in physiological and pathological processes *via* ferroptosis regulation. GPX4 uses glutathione (GSH) as an electron donor to reduce lipid peroxides, generating GSSG. Unlike other GPX family members, GPX4 acts within the lipid bilayer to directly reduce membrane-bound phospholipid hydroperoxides (PLOOH) to phospholipid alcohols (PL-OH). In contrast, GPX1 and GPX2 show high catalytic efficiency toward soluble substrates such as H_2_O_2_ and free fatty acid hydroperoxides (FA-OOH) but lack activity toward membrane-inserted PLOOH, rendering them unable to compensate for GPX4 loss in ferroptosis suppression. Under physiological conditions, GPX4 scavenges lipid peroxides to maintain redox homeostasis; its dysfunction leads to PLOOH accumulation, ferroptotic cell death, and tissue damage.

This mechanism places GPX4 at the intersection of redox sensing and signal transduction. By controlling the levels of hydroperoxide intermediates, GPX4 helps determine the final products of arachidonic acid metabolism and, in doing so, may influence platelet function and thrombotic processes (Sutherland et al., 2001) [Figure 5].

### 5.2 Roles in disease pathogenesis

#### 5.2.1 Cancer

GPX4 plays a context-dependent role in cancer. Its primary function is to suppress ferroptosis, thereby supporting tumor cell survival, proliferation, metastasis, and therapy resistance. In most cases, high GPX4 expression correlates with aggressive tumor behavior and poor prognosis, making its inhibition a promising therapeutic strategy. However, most evidence comes from preclinical models, including cell lines, xenografts, and retrospective clinical correlation studies.

Clinical correlative studies have consistently linked elevated GPX4 expression to aggressive tumor behavior and reduced patient survival across multiple solid malignancies, including hepatocellular, lung, breast, pancreatic, ovarian, thyroid, prostate, and oral squamous cell carcinomas (Li et al., 2024; Hori et al., 2024; Sha et al., 2021; Peng et al., 2019; Wu X. et al., 2022; Chen H. et al., 2023; Cheng L. et al., 2023; Zheng X. et al., 2024; Rao et al., 2024). In papillary thyroid carcinoma, GPX4 overexpression independently predicts shorter 5-year overall survival in retrospective cohort analyses (Sekhar et al., 2022). Genetic association studies have further identified the rs3746162 polymorphism in the GPX4 3′ UTR as an independent predictor of postoperative recurrence in non-muscle invasive bladder cancer (Ke et al., 2015). Mechanistically, cell-based studies indicate that GPX4’s antioxidant activity helps cancer cells counteract lipid peroxidation and evade ferroptosis. For instance, GPX4 knockdown in thyroid cancer cell lines suppresses proliferation and induces ferroptosis, and treatment with the GPX4 inhibitor RSL3 reduces viability and migratory capacity in these *in vitro* models (Chen H. et al., 2023; Sekhar et al., 2022).

The dependence of tumor cells on GPX4 varies considerably across tissue types, and this variation does not simply parallel GPX4 mRNA levels. A CRISPR-based genetic screen conducted in nearly 1,000 cancer cell lines revealed that the cell lines derived from thyroid, kidney, liver, hematopoietic, and nervous system tissues exhibit the strongest dependency on GPX4 and the selenoprotein synthesis pathway, whereas those from digestive tract, cervical, breast, and lung cancers are less dependent (Mao et al., 2021; Kinowaki et al., 2018). This differential dependency likely reflects lineage-specific differences in lipid metabolism and redox homeostasis. Supporting this observation, the same study found that the essentiality of SCD negatively correlates with that of GPX4 in these cell models: SCD generates monounsaturated fatty acids that resist peroxidation, thereby reducing the need for GPX4-mediated protection (Mao et al., 2021). This inverse relationship provides a mechanistic rationale that may inform tumor stratification strategies for GPX4-targeted therapy, although its predictive value remains to be tested in clinical settings.

GPX4 expression and activity are controlled at multiple levels, with both activators and repressors operating in a context-dependent manner. At the transcriptional level, studies in cancer cell lines have shown that CREB, PITX2, and the Wnt-regulated NR2F2 directly activate the GPX4 promoter, whereas KLF11, TNFSF11 (RANKL), and other factors repress its transcription (Wang et al., 2021; Liu W. et al., 2021; Zhao G. et al., 2023; Li et al., 2024; Wan et al., 2024). This regulatory balance determines basal GPX4 levels and sensitivity to ferroptosis inducers in these cell models. Post-transcriptionally, non-coding RNAs contribute to GPX4 regulation: circular RNAs, such as circ0060467 and circKIF4A, have been reported to sponge repressive microRNAs (miR-6805 and miR-1231), thereby elevating GPX4 protein levels and suppressing ferroptosis in cultured cancer cells (Tan et al., 2024; Chen W. et al., 2021); conversely, miR-324-3p directly targets GPX4 mRNA in cell-based assays (Hou et al., 2021). In addition, m^6^A methylation *via* the reader IGF2BP3 has been shown to enhance GPX4 mRNA stability in glioma cell lines (Deng et al., 2024). At the protein level, ubiquitination is a major determinant of GPX4 stability. In various cellular models, E3 ligases including TRIM21, FBXO31, and TRIM26 promote GPX4 degradation, whereas deubiquitinases such as OTUB1, OTUD5, USP10, and USP31 enhance its stability (Wang et al., 2023; Sun et al., 2023; Chu et al., 2023; Li et al., 2023; Ruan et al., 2025; Lian et al., 2024; Zhu et al., 2022; Ding et al., 2025). In gastric cancer, CST1 recruits OTUB1 to GPX4, enhancing GPX4 stability and suppressing ferroptosis, suggesting that disrupting this protein–protein interaction could be an indirect targeting strategy (Li et al., 2023). Kinases such as CKB and SGK2, as well as cytoskeletal proteins such as CK19, also modulate GPX4 stability (Wu et al., 2023; Cheng L. et al., 2023; Rao et al., 2024). Furthermore, as a selenoprotein, GPX4 depends on selenium supply; in glioblastoma, selenoprotein P (SeP) maintains GPX4 protein levels through local selenium bioavailability, and targeting this axis sensitizes tumors to GPX4 inhibitors (Zheng X. et al., 2024). The diversity of these regulatory nodes implies that resistance to direct GPX4 inhibitors may arise from multiple mechanisms, and combination approaches targeting specific nodes may be required.

Preclinically, direct GPX4 inhibitors such as RSL3 have been shown to induce ferroptosis in a range of cancer cell lines and xenograft models, an effect that is specifically rescued by ferrostatin-1 but not by caspase inhibitors, confirming its ferroptosis-specific nature (Sekhar et al., 2022). GPX4 inhibition has also been explored as a strategy to overcome acquired chemoresistance: clinical tissue analyses in ovarian cancer have shown that high co-expression of GPX4 and SLC7A11 correlates with platinum resistance and poor survival, and siRNA-mediated GPX4 silencing reduces resistance in corresponding cell lines (Wu X. et al., 2022; Lu et al., 2023); in preclinical models of lung cancer brain metastasis, a Wnt/NR2F2/GPX4 axis was found to drive acquired platinum resistance (Liu W. et al., 2021). Similarly, loss of the transcriptional repressor KLF11 relieves GPX4 repression in lung adenocarcinoma cell models, promoting chemoresistance, and restoring KLF11 downregulates GPX4 and increases sensitivity to cisplatin and pemetrexed *in vitro* (Zhao G. et al., 2023). Furthermore, ionizing radiation has been reported to upregulate GSTM3, which inhibits GPX4 and promotes ferroptosis in nasopharyngeal carcinoma cell lines, thereby enhancing radiosensitivity *in vitro* (Chen Y. et al., 2024); similar effects have been observed with hemin treatment in lung cancer cells (Almahi et al., 2022).

Despite these promising data, resistance to GPX4 inhibition remains a major barrier. Tumor cells rely on parallel ferroptosis defense pathways, including mitochondrial DHODH, which generates ubiquinol to inhibit lipid peroxidation. Concurrent inhibition of DHODH and GPX4 produces synergistic antitumor effects, offering a strategy to overcome resistance (Mao et al., 2021; Yao et al., 2023). Moreover, the tumor microenvironment adds another layer of complexity: GPX4 is required for the survival and effector function of anti-tumor CD8^+^ T cells (Chen S. et al., 2024). Thus, systemic GPX4 inhibition could inadvertently impair anti-tumor immunity, underscoring the need for cell-type-specific delivery systems. In summary, although GPX4 is a validated anticancer target, successful clinical translation will require addressing tumor heterogeneity, adaptive resistance, and immune-related toxicity through rational combination and targeted delivery.

#### 5.2.2 Neurodegenerative diseases

Ferroptosis has been implicated in the pathogenesis of various neurological disorders where oxidative stress and lipid peroxidation are prominent. A recurring observation is the downregulation of GPX4 in diseased tissues, suggesting its loss as a pathogenic event. However, the mechanisms leading to GPX4 dysfunction and the therapeutic implications differ substantially across diseases. In tissue samples from patients with multiple sclerosis (MS) and in the experimental autoimmune encephalomyelitis (EAE) mouse model, GPX4 mRNA levels are reduced in gray matter, correlating with the markers of lipid peroxidation and neuronal ferroptosis (Hu et al., 2019). In schizophrenia, analysis of *postmortem* brain tissue has shown that the endogenous retroviral element ERVW-1 negatively correlates with GPX4 and SLC3A2 expression, and mechanistic studies in cell models indicate that ERVW-1 directly represses GPX4 transcription, leading to neuronal ferroptosis (Zhang et al., 2024). These findings suggest an association between GPX4 loss and neuronal death in these disease contexts. The mechanisms underlying GPX4 dysfunction appear disease-specific. In PD, a potential mechanistic link involves the pathogenic protein DJ-1. Cell-based studies have shown that DJ-1, an oxidative stress-sensitive RNA-binding protein, binds to the GPX4 mRNA 5′ UTR and represses its translation; upon oxidative stress, DJ-1 dissociates, allowing increased GPX4 synthesis, whereas PD-associated DJ-1 mutants lack this regulatory capacity, which may contribute to neuronal vulnerability (Schriever et al., 2017). Although this provides an elegant molecular explanation, its *in vivo* relevance to PD pathogenesis and therapeutic potential are currently supported primarily by cell-based studies and await further validation in animal models. In contrast, the mechanism in schizophrenia is driven by transcriptional repression *via* ERVW-1, suggesting that restoring GPX4 function in different neurodegenerative conditions may require distinct intervention strategies (Zhang et al., 2024).

The potential of GPX4 pathway activation as a neuroprotective strategy is supported by studies on acute injury and drug-induced neurotoxicity. In intracerebral hemorrhage models, upregulating GPX4 expression reduces neuronal apoptosis, ferroptosis, and oxidative damage (Liu C. et al., 2024). In differentiated SH-SY5Y neuronal cells, the psychostimulant methamphetamine reduces GPX4 protein levels in a dose-dependent manner, correlating with decreased cell viability and increased ROS; selenium supplementation reverses this effect by restoring GPX4 expression, highlighting the role of selenium status in maintaining neuronal redox homeostasis (Barayuga et al., 2013). Despite these promising preclinical findings, the therapeutic implications for chronic neurodegenerative diseases, such as MS, PD, and schizophrenia, remain largely theoretical. A major barrier is the lack of safe and effective strategies to selectively upregulate GPX4 in affected neuronal populations without interfering with its essential functions in other tissues.

#### 5.2.3 Kidney diseases

GPX4 plays an important protective role in kidney diseases, particularly in AKI and chronic rejection. In I/R-induced AKI, ferroptosis of renal tubular epithelial cells (RTECs) is a central event leading to functional impairment, and GPX4 has been identified as a major protective factor in this process (Sun et al., 2023; Chu et al., 2023).

Selenium-binding protein 1 (SBP1) is downregulated during AKI and protects RTECs from ferroptosis by upregulating GPX4 (Zhao et al., 2024). The opposing actions of ubiquitination and deubiquitination tightly govern GPX4 stability in AKI, and this balance represents a potential therapeutic node. In animal models of I/R-induced acute kidney injury, the E3 ligase TRIM21 has been shown to promote ferroptosis by driving GPX4 degradation (Sun et al., 2023), whereas the deubiquitinase OTUD5 exerts a protective effect by stabilizing GPX4, although its own degradation *via* mTORC1-mediated autophagy during I/R undermines this protective axis (Chu et al., 2023). The therapeutic relevance of this regulatory axis is supported by preclinical findings that the JAK2 inhibitor fedratinib downregulates TRIM21, thereby preserving GPX4 levels and ameliorating renal injury in these models (Sun et al., 2023).

In renal fibrosis resulting from chronic allograft rejection, ferroptosis also contributes to disease progression. In a chronic renal dysfunction (CRD) model, TNF-α upregulates IRF1, which, together with ZNF350, forms a transcriptional repressor complex that binds to the GPX4 promoter and inhibits its transcription. This leads to ferroptosis in RTECs and secretion of pro-fibrotic factors, driving interstitial fibrosis (Zhang et al., 2022).

In diabetic nephropathy (DN), ferroptosis of RTECs is another key pathological event. Under high glucose conditions, the metalloproteinase DEP1 is upregulated and promotes ferroptosis through the inhibition of the GSH/GPX4 axis. High glucose directly downregulates GPX4 mRNA and protein expression, inducing ferroptosis in renal tubular epithelial cells. The natural compound platycodin D was shown to reverse this effect and upregulate GPX4, alleviating high glucose-induced ferroptosis and offering a potential strategy for diabetic nephropathy treatment (Lin et al., 2023).

Genetic studies have also indicated that GPX4 polymorphisms are associated with nephropathy risk in patients with type 1 diabetes in a sex-specific manner. In addition, proteomic analysis of urinary exosomes may offer a non-invasive approach for biomarker discovery in kidney disease, potentially including proteins such as GPX4 in the future (Gonzales et al., 2009).

#### 5.2.4 Liver diseases

GPX4 appears to contribute to liver homeostasis, and its dysfunction has been implicated in several liver diseases, particularly acute liver injury. In a lipopolysaccharide/D-galactosamine (LPS/D-GalN)-induced mouse model of acute liver failure (ALF), ferroptosis has been implicated as a driver of hepatocyte death. GPX4 and SLC7A11 were found to be downregulated in liver tissue in ALF patients and in the animal model. Mechanistic studies in cell lines and mouse models suggest a SIRT1–p53 axis: under ALF conditions, reduced SIRT1 expression and activity may lead to increased p53 acetylation and activation, which, in turn, may repress GPX4 transcription and promote lipid peroxidation and ferroptosis. Pharmacological activation of SIRT1 (e.g., with resveratrol) or genetic upregulation of SIRT1 was shown to deacetylate p53 and reduce its activity, with a corresponding increase in GPX4 expression and alleviation of liver injury, iron overload, and inflammation in these preclinical models (Zhou XN. et al., 2024). These observations point to a potential role for the SIRT1–p53–GPX4 axis in ALF and suggest that this axis may merit further exploration as a therapeutic target.

In non-alcoholic fatty liver disease, the E3 ligase TRIM59 has been shown to promote hepatocyte ferroptosis and hepatic steatosis by enhancing GPX4 ubiquitination and degradation (Zhang et al., 2023). In liver fibrosis, the mitophagy receptor FUNDC1 may facilitate hepatocyte ferroptosis and fibrosis progression through binding to GPX4 and mediating its mitochondrial translocation and autophagic degradation (Bi et al., 2024). In drug-induced liver injury, acetaminophen overdose was found to induce GPX4 carbonylation and proteasomal degradation in hepatocytes, leading to ferroptosis and liver necrosis (Ma et al., 2026). This mechanism may contribute to acetaminophen hepatotoxicity, and GPX4 stabilization has been suggested as a possible therapeutic strategy.

#### 5.2.5 Viral infection

The interaction between viral infection and the host GPX4 pathway is complex, with evidence pointing to opposing strategies that either exploit or suppress this antioxidant system to favor viral persistence. On the one hand, certain viruses appear to upregulate GPX4 to promote their own replication and survival. For instance, hepatitis C virus (HCV) infection induces GPX4 expression, which helps maintain the membrane fusion activity and infectivity of newly produced viral particles by controlling lipid peroxidation (Brault et al., 2016). Similarly, EBV upregulates GPX4 and SLC7A11 *via* the EBNA1–p62–Keap1–NRF2 axis, thereby enhancing resistance to ferroptosis and chemotherapy in nasopharyngeal carcinoma (Yuan et al., 2022). On the other hand, an alternative viral strategy involves the suppression of the GPX4 system, which may exacerbate host oxidative stress to facilitate viral replication. This is exemplified by the HIV Tat protein, which impairs GPX4 expression and activity, leading to glutathione depletion (Richard et al., 2001; Opalenik et al., 1998), and by the observation of GPX4 promoter hypermethylation in chronic hepatitis B (CHB), which may drive its transcriptional silencing. This epigenetic change appears particularly prominent during the immune tolerant phase and may facilitate viral persistence by inhibiting the cGAS–STING antiviral pathway (Su et al., 2024). In addition, hepatitis B virus surface antigen (HBsAg) has been reported to induce ferroptosis in testicular Sertoli cells, which may be relevant to infertility risk in male HBV carriers (Pan et al., 2024).

On the other hand, GPX4 may also play a role in the host antiviral innate immune response. The cGAS–STING pathway is central to cytosolic DNA sensing and antiviral immunity. Studies have suggested that redox homeostasis maintained by GPX4 is important for proper STING activation. When GPX4 function is compromised, accumulation of lipid peroxides may induce carbonylation of the STING protein, impairing its trafficking from the endoplasmic reticulum to the Golgi and thereby reducing STING signaling and type I interferon production. This could ultimately weaken host defense against DNA viruses (Chen Y. et al., 2025).

#### 5.2.6 Cardiovascular and metabolic diseases

GPX4 dysfunction has been linked to the pathology of various cardiovascular and metabolic diseases, many of which are accompanied by elevated systemic or local oxidative stress.

In preeclampsia, a hypertensive disorder specific to pregnancy, oxidative stress is considered a core pathogenic factor. Genetic case–control studies in the Chinese Han population have reported that the rs713041 polymorphism in the GPX4 gene is associated with susceptibility to preeclampsia, with the C allele conferring a higher risk across mild-, severe-, and early-onset groups, suggesting that this locus may represent a genetic risk factor in this population (Peng et al., 2016); a second study has also reported an association between this locus and preeclampsia (Chen et al., 2020). In cell-based mechanistic studies, the DJ-1 protein has been shown to inhibit ferroptosis in trophoblast cells through upregulation of the Nrf2/GPX4 pathway, which may contribute to its protective role (Liao et al., 2022).

In atherosclerosis, lipid peroxidation and macrophage dysfunction are key events. The long non-coding RNA lnc-MRGPRF-6:1 has been found to promote ferroptosis in oxidized low-density lipoprotein (ox-LDL)-stimulated macrophages by inhibiting GPX4, suggesting a possible role in atherosclerotic plaque formation (You et al., 2023). Endothelial dysfunction is an early event in atherosclerosis, and GPX4 expression and activity in endothelial cells appear to be regulated by polyunsaturated fatty acids and inflammatory cytokines (Sneddon et al., 2003).

The rs713041 polymorphism has also been studied for its potential effects on endothelial cell function. In one functional study, human umbilical vein endothelial cells (HUVECs) carrying the TT genotype showed enhanced monocyte adhesion compared with cells with the CC genotype, which appeared to be related to higher VCAM-1 expression under pro-inflammatory conditions, such as arachidonic acid treatment. Notably, this effect seemed to be influenced by selenium status: the hyper-adhesive phenotype associated with the TT genotype was more pronounced under selenium-deficient conditions, and selenium supplementation partially reversed it. These findings suggest that this SNP may affect vascular function through interactions with dietary factors, such as selenium and fatty acids (Polonikov et al., 2012).

In metabolic diseases, GPX4 has also drawn attention in the context of gestational diabetes mellitus (GDM). circHIPK3 has been reported to promote ferroptosis in GDM through the regulation of GPX4 DNA methylation (Jiang et al., 2023). SIRT3 deficiency was found to confer resistance to autophagy-dependent ferroptosis by inhibiting the AMPK/mTOR pathway and increasing GPX4 levels (Han et al., 2020). In addition, metformin, a commonly used hypoglycemic agent, has been shown to induce ferroptosis in breast cancer cells through the miR-324-3p/GPX4 axis, revealing a possible mechanism for its antitumor effects and illustrating the crosstalk between metabolic regulation and ferroptosis (Hou et al., 2021).

#### 5.2.7 Autoimmune diseases

GPX4 has been studied in several autoimmune conditions. Its expression and activity are frequently reduced in autoimmune settings, and this loss has been associated with ferroptosis in immune cells and parenchymal cells (Li et al., 2021a; Alli et al., 2023; Tao et al., 2024; Hu et al., 2019).

In SLE, autoantibodies and type I interferon enhance the binding of the transcriptional repressor CREMα to the GPX4 promoter, suppressing GPX4 transcription in neutrophils. This leads to lipid peroxide accumulation, ferroptosis, and the release of neutrophil extracellular traps, which, in turn, stimulate plasmacytoid dendritic cells to produce more interferon-α (Li et al., 2021a). In lupus nephritis, GPX4 downregulation in renal tubular epithelial cells has been linked to ferroptosis and tubulointerstitial pathology (Alli et al., 2023). GPX4 protein levels in peripheral blood mononuclear cells from SLE patients correlate inversely with disease activity scores (Tao et al., 2024).

In rheumatoid arthritis (RA), GPX4 appears to exert cell-type-dependent effects. In synovial fibroblasts, GPX4 is upregulated *via* pathways such as PI3K–AKT–mTOR signaling, which is associated with resistance to ferroptosis and synovial hyperplasia (Cheng et al., 2022). In macrophages, GPX4 loss is linked to ferroptosis and inflammatory responses (Liu Y. et al., 2024). Natural compounds have been reported to ameliorate experimental arthritis by restoring GPX4 expression through the Nrf2 pathway (Zhou et al., 2026).

In Sjögren’s syndrome, interferon-γ suppresses the system Xc^−^–GSH–GPX4 axis in salivary gland epithelial cells *via* JAK–STAT1 signaling, inducing ferroptosis and impairing secretory function (Cao et al., 2023). Downregulated GPX4 in these cells also affects the lipid ROS/pSTAT4/AQP5 axis (Zhou J. et al., 2024). In multiple sclerosis, inflammatory stress in neurons triggers non-canonical STING signaling, which promotes autophagic degradation of GPX4 (Hu et al., 2019). GPX4 levels are reduced in central nervous system tissues from patients and correlate with disease severity (Stojkovic et al., 2024).

GPX4 dysfunction has also been implicated in psoriasis (Vats et al., 2025), IgA nephropathy (Wu J. et al., 2022), autoimmune hepatitis (Zhu et al., 2021), and type 1 diabetes (Cao et al., 2024), where ferroptosis contributes to epithelial damage, mesangial cell injury, hepatocyte death, and β-cell destruction, respectively. Pharmacological strategies aimed at stabilizing or upregulating GPX4, such as selenium supplementation (Be et al., 2005) or Nrf2 activation (Weng et al., 2025), are being explored. However, the context-dependent and cell-type-specific roles of GPX4 require further investigation to guide appropriate intervention strategies.

#### 5.2.8 Other diseases

GPX4 dysfunction has also been explored in several other diseases involving oxidative stress and inflammation.

##### 5.2.8.1 Osteoarthritis (OA)

Ferroptosis is thought to contribute to chondrocyte death and cartilage degeneration in OA. In temporomandibular joint osteoarthritis (TMJOA), analyses of clinical synovial tissue and fibroblast-like synoviocytes from patients have shown elevated ferroptosis markers (iron ions and 8-OHdG) and reduced antioxidant activity (GSH content and the GSH/GSSG ratio). GPX4 mRNA and protein were markedly downregulated, while ACSL4 was increased, and SLC3A2 decreased in these samples. In cell-based experiments, GPX4 downregulation was found to promote synovial cell ferroptosis, and ferroptosis inhibitors appeared to protect cells and slow disease progression *in vitro* (Pang et al., 2024). The senescence marker p21 was reported to protect OA chondrocytes from ferroptosis by stabilizing the GPX4 protein (Zheng Z. et al., 2024).

##### 5.2.8.2 Pulmonary diseases

In COPD, cigarette smoke exposure may induce macrophage ferroptosis through the USP7/GPX4 axis (Ruan et al., 2025). Genetic susceptibility in COPD appears to involve other pathways as well. In a case–control study from Xuanwei, China, where indoor coal combustion contributes to high COPD rates, PTEN variants (rs701848 and rs1903858) were associated with reduced disease risk (Hosgood et al., 2009). Since PTEN negatively regulates AKT signaling and is linked to airway inflammation, this suggests that PTEN/AKT pathway dysregulation, in addition to ferroptosis, may play a role in COPD, particularly under prolonged pollutant exposure (Hosgood et al., 2009). However, this latter association derives from a single genetic study and requires replication in independent populations before firm conclusions can be drawn. In IPF, GPX4 expression was lower in lung tissue, and lipid peroxidation products were increased; GPX4 deficiency worsened bleomycin-induced fibrosis in mice, pointing to a protective role for GPX4 against pulmonary fibrosis (Tsubouchi et al., 2019).

##### 5.2.8.3 Pancreatitis

The GPX4 variant rs713041 may interact with smoking to affect acute pancreatitis risk (Ściskalska and Milnerowicz, 2022). Ferroptosis has also been implicated in acute pancreatitis, and copper chelators were found to reduce disease severity in experimental models by stabilizing GPX4 (Xue et al., 2023).

##### 5.2.8.4 Intervertebral disc degeneration (IVDD)

Mechanical overload appears to trigger ferroptosis in nucleus pulposus cells. Excessive mechanical stress activates Piezo1, increasing intracellular Ca^2+^ levels, which may impair GPX4 function and induce ER stress. This seems to create a feedback loop that worsens GPX4 dysfunction and lipid peroxidation. Selenium supplementation upregulated selenoprotein K and GPX4, forming a protective axis that reduced ER stress and calcium levels, thereby protecting cells from ferroptosis and slowing disc degeneration (Jia et al., 2024).

##### 5.2.8.5 Sepsis

GPX4 has emerged as a possible ferroptosis-related biomarker in pediatric sepsis. Its expression correlated negatively with neutrophils and PD-L1 and positively with CD8^+^ T cells, suggesting a role in sepsis-related immunosuppression. In animal models, GPX4 protein levels were reduced in multiple organs (heart, lung, liver, and kidney) during bacterial sepsis (Qu et al., 2023). In sepsis-associated acute lung injury, adipose-derived stem cell exosomes delivering miR-125b-5p were found to target Keap1 and relieve its inhibition of Nrf2, leading to increased GPX4 expression and reduced ferroptosis in pulmonary microvascular endothelial cells. This was associated with less lung damage and improved survival in animal models (S et al., 2023).

## 6 Therapeutic strategies targeting GPX4 and perspectives

With a better understanding of how GPX4 influences cell fate, particularly through ferroptosis regulation, interest in targeting this enzyme for therapeutic purposes has grown, especially in oncology. Current strategies can be grouped into two main categories: direct inhibition of GPX4 catalytic activity and indirect modulation of its function. Key approaches are outlined in Table 5.

**TABLE 5:** Summary of therapeutic strategies targeting GPX4.

Category	Name	Content/function	Reference
Direct inhibition	RSL3/ML-162	Direct covalent binding and irreversible inhibition of GPX4 activity, leading to lipid peroxide accumulation, thereby inducing ferroptosis in various cancer cells and sensitizing tumors to chemotherapy and radiotherapy.	Shin et al. (2018), Liu et al. (2021a), Huan et al. (2024), and Li et al. (2021b)
Indirect inhibition	Nrf2 inhibitors (e.g., trigonelline)	Combined with GPX4 inhibitors to suppress the Nrf2 signaling pathway, prevent compensatory antioxidant responses, and overcome resistance to GPX4 inhibitors.	Shin et al. (2018)
​	Orexin-A	Depletes GPX4 and targets NFE2L2 (Nrf2) in glioblastoma, synergistically inducing ferroptosis.	Huan et al. (2024)
​	TGF-β1 signaling activation	Suppresses SLC7A11 expression, reduces glutathione synthesis, and sensitizes hepatocellular carcinoma cells to GPX4 inhibitors.	Kim et al. (2020)
​	CKB inhibitors	Targeting CKB reduces S104 phosphorylation of GPX4, promoting its degradation *via* the CMA pathway and indirectly downregulating GPX4 levels.	Wu et al. (2023)
​	mTOR inhibitors (e.g., rapamycin)	Inhibition of the mTOR pathway induces GPX4 degradation, revealing a link between metabolic signaling and ferroptosis regulation.	Liu et al. (2021b)
​	Pro-degradative E3 ligase activation (TRIM21, TRIM59, and FBXO31)	Activates cognate E3 ubiquitin ligases to promote ubiquitination and degradation of GPX4, thereby inducing ferroptosis.	Sun et al. (2023), Zhu et al. (2022), and Pickel et al. (2019)
​	Deubiquitinase inhibitors (OTUD5, OTUB1, and USP7)	Inhibits cognate deubiquitinases to accelerate GPX4 ubiquitination and degradation, inducing ferroptosis.	Liu et al. (2023), Li et al. (2023), and Ruan et al. (2025)
Activation/enhancement	Stabilizing E3 ligase activation (LUBAC and TRIM26)	Activates specific E3 ligases to stabilize GPX4 *via* non-degradative ubiquitin chains, thereby inhibiting ferroptosis.	Tuo et al. (2026) and Wang et al. (2023)
​	ADSC exosomes (miR-125b-5p)	Exosome-mediated delivery of miR-125b-5p to target cells activates the Nrf2 pathway by repressing Keap1, upregulating GPX4 expression and inhibiting ferroptosis to protect tissues (e.g., acute lung injury).	S et al. (2023)
​	SIRT1 activators (e.g., resveratrol)	Activates SIRT1, which deacetylates and inhibits p53, relieving its transcriptional repression of GPX4, thereby upregulating GPX4 and alleviating ferroptosis-mediated liver injury.	Zhou et al. (2024a)
​	HSP90 inhibitors (e.g., 17-AAG)	Inhibiting HSP90 blocks chaperone-mediated autophagy (CMA), thereby stabilizing GPX4 and antagonizing ferroptosis induction.	Zhu et al. (2025)

### 6.1 GPX4 inhibitors and ferroptosis inducers

Direct inhibition of GPX4 enzymatic activity remains the most straightforward approach to triggering ferroptosis. Until now, a number of small-molecule inhibitors that covalently modify the selenocysteine residue in the GPX4 active site have been developed. Among them, Ras-selective lethal 3 (RSL3) and ML-162 are the most extensively characterized canonical GPX4 inhibitors. These compounds irreversibly suppress GPX4 function, leading to substantial accumulation of lipid peroxides and efficient ferroptosis induction across diverse cancer cell types (Shin et al., 2018). For example, RSL3 has been shown to effectively induce ferroptosis in preclinical models of thyroid cancer, acting independently of apoptosis, with this effect being reversed by the specific ferroptosis inhibitor ferrostatin-1 (Sekhar et al., 2022). In preclinical settings, these inhibitors have demonstrated significant antitumor activity in multiple tumor models, including head and neck cancer, ovarian cancer, and glioblastoma (Shin et al., 2018; Huan et al., 2024; Li D. et al., 2021). In addition, they have been reported to sensitize brain metastases of lung cancer to platinum-based chemotherapy, offering a possible strategy to overcome acquired resistance (Liu W. et al., 2021). More recently, advances in targeted protein degradation have led to the development of brain-penetrant GPX4 PROTACs, which show potent anti-glioblastoma activity in orthotopic mouse models (Li et al., 2026). In parallel, antibody–drug conjugates that link GPX4 inhibitors to tumor-specific antigens have been found to improve tumor selectivity and reduce systemic toxicity in preclinical studies (Khan et al., 2025).

Nevertheless, tumor heterogeneity and adaptive responses give rise to resistance against GPX4 inhibitors, which remains a major challenge for clinical translation. A key mechanism underlying this resistance involves activation of the nuclear factor erythroid 2-related factor 2 (Nrf2) signaling pathway. As a central regulator of cellular antioxidant defense, Nrf2 upregulates multiple antioxidant genes, including those involved in glutathione biosynthesis, thereby partially compensating for GPX4 loss and dampening ferroptotic responses (Shin et al., 2018). In light of this, combination strategies have been proposed to counteract resistance. For instance, the Nrf2 inhibitor trigonelline has been found to reverse RSL3 resistance in head and neck cancer cells (Shin et al., 2018). In addition, certain natural and synthetic compounds have been shown to act synergistically with GPX4 inhibitors. Orexin-A, for example, not only depletes GPX4 but also targets NFE2L2 (Nrf2) in glioblastoma, thus cooperatively promoting ferroptosis (Huan et al., 2024). Another promising approach is to interfere with the upstream substrate supply for GPX4. Transforming growth factor-β1 (TGF-β1) has been reported to repress SLC7A11 expression, a key subunit of the cystine/glutamate antiporter system x_c^−^, thereby reducing cystine uptake and glutathione synthesis and rendering hepatocellular carcinoma cells particularly susceptible to GPX4 inhibition. This observation provides a mechanistic basis for combining TGF-β pathway blockade with GPX4 targeting (Kim et al., 2020).

### 6.2 Indirect targeting strategies

Beyond direct inhibition of GPX4 enzymatic activity, indirect modulation of GPX4 function by interfering with its upstream regulatory network or functionally cooperative pathways represents an alternative strategy.

#### 6.2.1 Kinases and phosphatases

Creatine kinase B (CKB) acts as a protein kinase that phosphorylates GPX4 at Ser104, preventing its interaction with the chaperone protein HSC70 and thereby inhibiting CMA-dependent GPX4 degradation and enhancing its stability (Wu et al., 2023). Moreover, the canonical mTOR signaling pathway is also linked to GPX4 stability. The mTOR inhibitor rapamycin induces GPX4 degradation, uncovering a direct connection between metabolic signaling and ferroptosis regulation (Liu Y. et al., 2021). In thyroid cancer, GPX4 inhibition directly leads to inactivation of the mTOR pathway, suggesting a feedback regulatory loop between GPX4 and mTOR (Sekhar et al., 2022). These findings indicate that targeting upstream kinases such as CKB or mTOR can indirectly regulate GPX4 abundance. Similar to the central role of the protein phosphatase PP1 in key processes including cell division (Capalbo et al., 2019), targeting kinases or phosphatases that control the phosphorylation status of GPX4 represents a potentially precise therapeutic intervention.

#### 6.2.2 E3 ligases and deubiquitinases

The therapeutic logic for targeting E3 ligases and DUBs is defined by a central principle: clinical outcomes depend on whether the target stabilizes or destabilizes GPX4. Thus, E3 ligases that promote GPX4 degradation (e.g., TRIM21, TRIM59, and FBXO31) are attractive targets for ferroptosis induction in oncology, whereas those that stabilize GPX4 (e.g., HOIP and TRIM26) represent nodes for sensitization (Tuo et al., 2026; Wang et al., 2023; Sun et al., 2023; Zhang et al., 2023; Zhu et al., 2022). However, this approach faces a major caveat: the effect of modulating these enzymes is highly context-dependent. The same ligase that suppresses tumor progression could, in a different setting, exacerbate renal injury, as observed with TRIM21 in ischemia–reperfusion injury (Sun et al., 2023). On the deubiquitination side, DUBs such as OTUD5 and OTUB1, which protect GPX4 from degradation, present a mirror-image opportunity for therapeutic intervention (Liu et al., 2023; Chu et al., 2023). Notably, the stabilizing effect of OTUB1 on GPX4 depends on its interaction with the tumor-promoting protein CST1 in gastric cancer, suggesting that disrupting this specific protein–protein interface could represent a more tumor-selective indirect strategy for GPX4 inhibition, potentially circumventing the broader toxicity associated with systemic DUB blockade (Li et al., 2023). Considering the involvement of TRIM family proteins in multiple cancers, including TRIM67 in oligodendroglioma and TRIM17 in gastric cancer (Demirdizen et al., 2023; Shen et al., 2023), targeting these GPX4-regulating E3 ligases or DUBs has become an area of growing interest.

In addition, modulating upstream non-coding RNAs that regulate GPX4 expression, such as circKIF4A which is highly expressed in thyroid cancer, offers another avenue for indirectly inducing ferroptosis (Chen W. et al., 2021). The pairing between E2 and E3 enzymes is relatively specific and can be precisely influenced by phosphorylation, which may offer druggable opportunities. Studies on the GID/CTLH E3 ligase and its dedicated E2 enzyme UBE2H have revealed that multi-site phosphorylation within the C-terminal extension of the E2 enzyme enables multivalent anchoring to the basic region of the E3 complex. Such phosphorylation-dependent interactions not only determine the specificity of E2–E3 pairing but also stabilize the catalytic conformation to facilitate ubiquitination (Chrustowicz et al., 2024). This finding highlights the role of post-translational modifications in defining E2–E3 partnerships and supports the feasibility of targeting these interactions. Indeed, chemical probes have been developed that specifically disrupt the N-terminal acetylation-driven interaction between the E2 enzyme UBE2M and the E3 ligase subunit DCN1, thereby effectively inhibiting the downstream NEDD8 conjugation pathway (Scott et al., 2017). This proof-of-concept study confirms that post-translational modification-dependent protein–protein interactions are pharmacologically tractable and provides a basis for developing agents that modulate GPX4 ubiquitination more specifically.

As an example, in a murine model of acute kidney injury, the JAK2 inhibitor fedratinib has been shown to stabilize GPX4 by downregulating the E3 ligase TRIM21, thereby exerting renoprotective effects and illustrating the feasibility of targeting upstream pathways to modulate E3 ligase activity (Sun et al., 2023). Furthermore, the large Cullin–RING ligase (CRL) family may also participate in this regulatory network, potentially broadening the range of targets for further exploration (Kouranti et al., 2022).

#### 6.2.3 Exosome-mediated delivery of regulatory molecules to enhance GPX4 function

In contrast to strategies aimed at inhibiting GPX4 for cancer therapy, boosting GPX4 expression and function represents a major protective approach in tissue injury diseases. Exosomes, as natural nanocarriers, provide a promising delivery platform. In a model of sepsis-induced acute lung injury, exosomes secreted by adipose-derived stem cells (ADSCs) deliver miR-125b-5p to pulmonary microvascular endothelial cells. miR-125b-5p targets and represses Keap1, relieving its inhibition of Nrf2, thereby activating Nrf2 and upregulating its downstream target GPX4. This strategy of exogenously elevating GPX4 *via* miRNA effectively inhibits endothelial ferroptosis and alleviates lung injury, opening new avenues for exosome-based tissue-protective therapy (S et al., 2023).

#### 6.2.4 Other regulatory factors

Sirtuin 1 (SIRT1), a well-known deacetylase, has also been linked to GPX4 regulation. In a model of acute liver failure, the activation of SIRT1, for instance, by resveratrol, leads to deacetylation and inhibition of the transcription factor p53, which, in turn, relieves p53-mediated transcriptional repression of GPX4. This results in increased GPX4 expression and attenuation of ferroptosis-related liver injury (Zhou XN. et al., 2024). In a similar context, the sphingosine-1-phosphate (S1P) signaling pathway has been reported to upregulate GPX4 and mitigate radiation-induced ferroptosis (Zhao J. et al., 2023). Together, these observations suggest that the acetylation status of GPX4 or its upstream regulators may be modulated to indirectly influence GPX4 function.

#### 6.2.5 Molecular chaperones and the CMA pathway

Although degrading GPX4 is often desirable in cancer therapy, targeting its upstream regulators has revealed complex functional interactions. For example, the molecular chaperone HSP90 stabilizes numerous oncoproteins, and its inhibitors (e.g., 17-AAG) are established anti-cancer agents. However, HSP90 plays a dual role in ferroptosis. HSP90 positively regulates CMA, a major pathway for GPX4 degradation. Consequently, inhibiting HSP90 blocks CMA activity, unexpectedly stabilizing GPX4 and suppressing ferroptosis induced by erastin and other inducers (Zhu et al., 2025). This finding underscores the need to caution against potential antagonistic effects when combining HSP90 inhibitors with ferroptosis inducers for anti-cancer therapy.

#### 6.2.6 The DHODH pathway

Dihydroorotate dehydrogenase (DHODH) is a mitochondrial inner membrane enzyme that participates in *de novo* pyrimidine synthesis. A previous study identified an additional function of DHODH in suppressing ferroptosis. Through its enzymatic activity, DHODH reduces mitochondrial coenzyme Q (CoQ) to ubiquinol (CoQH_2_), which acts as a radical-trapping antioxidant and specifically inhibits mitochondrial lipid peroxidation (Cao J. et al., 2025). This pathway appears to operate in parallel with the cytoplasmic GPX4 and FSP1 systems, alongside mitochondrial GPX4 (GPX4mito).

When DHODH is inhibited, for example, by brequinar, cancer cells show increased mitochondrial lipid peroxidation and become more dependent on GPX4. In tumor cells with low GPX4 expression, DHODH inhibition alone may be sufficient to trigger ferroptosis; in cells with higher GPX4 levels, combining DHODH and GPX4 inhibition produces a synthetic lethal effect. These observations point to the existence of a mitochondrial ferroptosis defense mechanism and suggest that combinatorial strategies may be worth exploring for tumors that do not respond well to GPX4 inhibitors alone (Liu Y. et al., 2026; Zhang et al., 2026).

#### 6.2.7 The selenocysteine biosynthesis pathway

**FIGURE 6:**
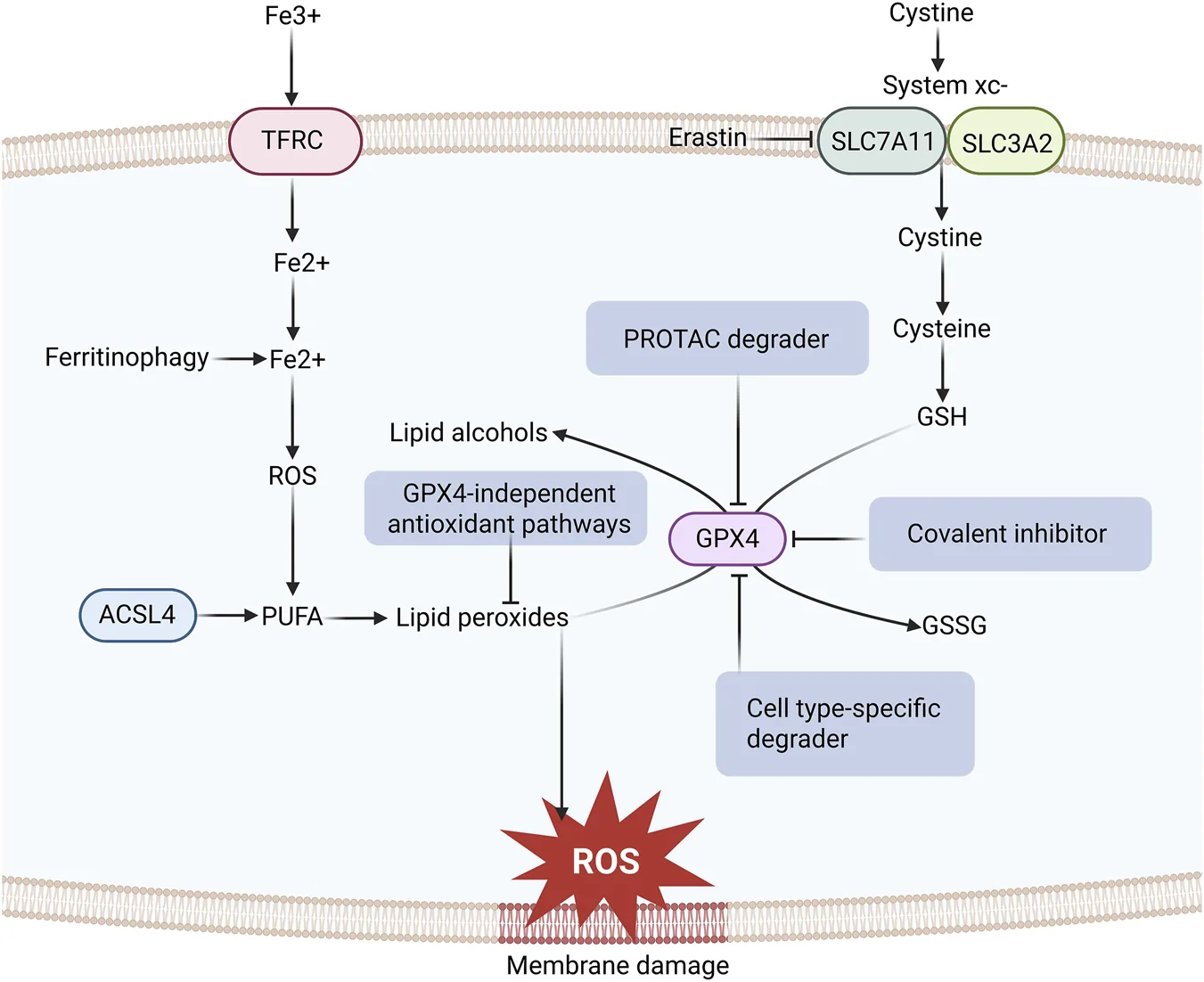
Schematic of GPX4-centered ferroptosis regulation and targeted therapeutic strategies. This diagram illustrates the core ferroptosis network centered on GPX4, covering iron metabolism, the system xc⁻‐GSH substrate axis, and multiple GPX4-targeting modalities including covalent inhibitors, PROTAC degraders and cell type-specific degraders. GPX4 inactivation drives lipid peroxide accumulation and membrane damage, while parallel GPX4-independent antioxidant pathways may contribute to therapeutic resistance.

GPX4 is a selenoprotein, and its biosynthesis and function rely on a functional Sec incorporation machinery. Synteny analysis has indicated that GPX4 is functionally linked to all known components of the Sec biosynthesis pathway, as well as to another selenoprotein, TXNRD1 (Mao et al., 2021). This connection increases the possibility that targeting key enzymes in the Sec biosynthesis pathway could impair GPX4 synthesis at an early stage and thereby indirectly induce ferroptosis (Figure 6).

This diagram illustrates the core ferroptosis network centered on GPX4, covering iron metabolism, the system xc^−^–GSH substrate axis, and multiple GPX4-targeting modalities, including covalent inhibitors, PROTAC degraders, and cell type-specific degraders. GPX4 inactivation drives lipid peroxide accumulation and membrane damage, while parallel GPX4-independent antioxidant pathways may contribute to therapeutic resistance.

## 7 Challenges and future directions

Over the past decade, progress in GPX4 biology has positioned it as a target of interest for several human diseases. Studies on its transcriptional regulation, post-translational modifications, and roles in disease contexts have provided a basis for considering GPX4-directed therapeutic strategies. Recent reports have described additional modifications of GPX4, including palmitoylation and arginine methylation, as well as immunomodulatory effects associated with extracellular GPX4 and structural insights into its neuroprotective functions (Huang et al., 2025; Zhou L. et al., 2025; Fan et al., 2025; Liu J. et al., 2026; Lorenz et al., 2026). These observations have extended the current understanding of GPX4 biology. At the same time, moving from mechanistic insights and preclinical findings to clinical applications faces several challenges. These include the complexity of its regulatory networks, limitations in druggability, context-dependent functional outcomes, resistance mechanisms, and the absence of validated biomarkers. This section outlines these scientific and translational issues and discusses possible future directions based on recent developments, with the aim of providing a conceptual reference for further work on GPX4-targeted interventions.

### 7.1 Core challenges in GPX4-targeted therapy and translational application

#### 7.1.1 Complexity of the GPX4 post-translational modification network and dilemmas in target specificity

Recent studies have expanded the understanding of GPX4 regulation beyond the transcriptional level and the ubiquitin–proteasome pathway. Multiple post-translational modifications, including palmitoylation, arginine methylation, phosphorylation, lactylation, and O-GlcNAcylation, have been reported to influence GPX4 protein stability, enzymatic activity, subcellular localization, and protein–protein interactions (Huang et al., 2025; Zhou L. et al., 2025; Hwang and Henry, 2025; Qian Z. et al., 2025; Zhou et al., 2025c). These modifications together form a complex regulatory network.

For example, S-palmitoylation mediated by ZDHHC8 and depalmitoylation by APT2 have been shown to bidirectionally regulate GPX4 stability and ferroptosis sensitivity (Huang et al., 2025; Zhou L. et al., 2025; Hwang and Henry, 2025). PRMT5-mediated symmetric dimethylation of GPX4 at Arg152 prolongs its half-life and has been associated with ferroptosis resistance in tumors (Fan et al., 2025). Phosphorylation of GPX4 at Ser45 by STK38 enhances its stability by blocking chaperone-mediated autophagy. In addition, lactylation, O-GlcNAcylation, and deubiquitination have also been implicated in GPX4 regulation (Qian Z. et al., 2025; Zhou et al., 2025c).

Several issues, however, remain to be addressed. The crosstalk among different modifications, such as the interplay between ubiquitination and palmitoylation or between methylation and phosphorylation, is not yet well understood. The regulatory effects of these modifications also appear to be cell-type- and tissue-specific; the same modification may have opposing effects in tumor cells *versus* normal cells or across different lineages. For instance, ZDHHC8-mediated palmitoylation of GPX4 contributes to ferroptosis resistance in cancer cells, but its physiological roles in normal cardiomyocytes and neurons remain unclear, increasing the possibility of off-target effects in normal tissues when modifying enzymes are targeted (Huang et al., 2025; Zhou L. et al., 2025; Xu et al., 2025a). Furthermore, many of these modifications appear to maintain basal GPX4 stability and redox balance under physiological conditions and may become dysregulated in disease states. At present, it remains difficult to selectively target pathological modifications without interfering with normal GPX4 function.

#### 7.1.2 Druggability bottlenecks and delivery system barriers for GPX4 targeting

Current drug development targeting GPX4 remains largely at the preclinical stage, and suboptimal druggability continues to be a major barrier to clinical translation. Classical GPX4 inhibitors such as RSL3 and ML162 are covalent compounds that show potent activity *in vitro*, but their clinical applicability is limited by poor water solubility, insufficient metabolic stability, low bioavailability, and off-target toxicity associated with nonspecific covalent binding.

Targeted protein degradation approaches, including proteolysis-targeting chimeras (PROTACs), autophagy-targeting chimeras (AUTACs), and lysosome-targeting chimeras (LYTACs), have been explored as alternative strategies for GPX4 modulation. As an example, GPX4-AUTACs have been developed to promote selective autophagic degradation of GPX4 through the TRAF6–p62 axis and have demonstrated ferroptosis induction at lower doses in experimental settings (Yang et al., 2025; Gong et al., 2025). Nevertheless, these degraders still face challenges, such as limited membrane permeability, inadequate *in vivo* stability, low tumor accumulation, and unfavorable pharmacokinetic properties, and their application remains confined to cellular and animal models.

Dual-targeting agents also present difficulties in balancing activities. The first dual PCBP1/2 and GPX4 inhibitor acevaltrate has been reported to synergistically induce ferroptosis in tumors, although further optimization is needed to improve activity matching and metabolic stability *in vivo* (Yu et al., 2025).

Delivery systems add another layer of complexity to druggability and safety considerations. GPX4 is required for normal embryonic development, and systemic inhibition may cause damage to tissues that depend heavily on GPX4, including the kidney, nervous system, and reproductive system. Therefore, achieving tissue- or cell-specific delivery appears to be an important prerequisite for safer GPX4-targeted therapy.

Several strategies have been explored, including mitochondria-specific GPX4 inhibitors, tumor microenvironment-responsive nanocarriers, and exosome-based delivery systems (Xiao et al., 2025). However, a number of issues remain. Many nanocarriers are cleared rapidly by the reticuloendothelial system, limiting their accumulation at target sites. Targeting efficiency is also suboptimal in some cases, with limited penetration into solid tumors and poor blood–brain barrier crossing, which restricts their use in central nervous system disorders and deep-seated tumors. In addition, immunogenicity and safety concerns persist as viral vectors and certain nanomaterials may elicit immune responses.

#### 7.1.3 Bidirectional effects of GPX4 intervention and systemic safety risks

As a regulator of cellular redox homeostasis, GPX4 appears to play opposing roles in different diseases and cell types, which complicates decisions about therapeutic direction. To interpret this heterogeneity, we propose a two-axis framework: disease context, which discusses malignancy *versus* acute or chronic tissue injury, and cell-type specificity, which discusses tumor cells *versus* immune or stromal cells.

In malignant tumors, GPX4 is often overexpressed and contributes to proliferation, drug resistance, and immune evasion by suppressing ferroptosis; in these contexts, inhibiting GPX4 to induce ferroptosis has been proposed. In contrast, conditions such as myocardial ischemia–reperfusion injury, acute kidney injury, neurodegenerative diseases, and organ fibrosis have been associated with reduced GPX4 function and ferroptosis-mediated tissue damage, suggesting that stabilizing or activating GPX4 may be protective (Wang et al., 2025a; Ye Z. et al., 2025; Liu et al., 2025a; Shi et al., 2025; Shahid et al., 2025). This bidirectionality underscores the need for precise disease and cell type specificity as an incorrect intervention strategy could potentially worsen disease outcomes.

Systemic safety presents an additional challenge. Global Gpx4 knockout is embryonically lethal in mice, and tissue-specific knockout models have shown that renal tubular epithelial cells, neurons, germ cells, and cardiomyocytes are particularly sensitive to GPX4 loss. Impaired GPX4 function has been linked to male infertility, renal tubular necrosis, neurodegeneration, and myocardial injury (Azuma et al., 2022; Tian et al., 2025).

Even with tumor-targeted delivery, immune-related safety and efficacy concerns remain. One recent study reported that extracellular GPX4 released by ferroptotic tumor cells can bind the ZP3 receptor on dendritic cells and suppress type I interferon production through the cAMP–PKA pathway, thereby dampening anti-tumor innate and adaptive immunity. This effect was found to reduce the efficacy of GPX4 inhibitors and may even facilitate immune escape (Liu J. et al., 2026).

In addition, functional heterogeneity of GPX4 across cell types within the tumor microenvironment adds further complexity. GPX4 knockdown has been reported to inhibit M2 macrophage polarization in the gastric cancer microenvironment, exerting anti-tumor effects (Xu et al., 2025b), whereas macrophage-specific GPX4 deletion has been shown to improve obesity-associated insulin resistance. These observations suggest that modulating GPX4 in non-tumor cells may confer additional benefits or risks. At present, strategies that selectively target GPX4 in tumor cells while sparing other cells in the microenvironment remain limited. This functional diversity complicates simple “activate or inhibit” decisions. We propose interpreting these opposing roles through two practical lenses. First, disease context: in malignancies where GPX4 is hijacked for survival, inhibition is logical; in acute injuries or neurodegeneration where its loss drives pathology, stabilization is preferred. Second, cell-type specificity: GPX4 loss in tumor cells may be therapeutic, but loss in immune effector cells could be detrimental. This framework provides a rationale for designing cell- and disease-specific interventions and for interpreting seemingly conflicting findings.

#### 7.1.4 Diverse resistance mechanisms and heterogeneous clinical responses

Adaptive resistance of tumor cells remains an obstacle to the clinical efficacy of GPX4-targeted therapy. Identified resistance mechanisms include compensatory pathway activation, transcriptional reprogramming, aberrant post-translational modifications, and tumor microenvironment crosstalk, reflecting considerable diversity and complexity.

Compensatory activation of parallel ferroptosis defense pathways appears to be a prominent mechanism of primary resistance. Pathways such as FSP1–CoQ10, DHODH–ubiquinone, and GCH1–BH4, which function independently of GPX4, have been found to be upregulated upon GPX4 inhibition and may attenuate its ferroptosis-inducing effects. The FSP1–CoQ10 axis operates at the plasma membrane, where it reduces ubiquinone (CoQ_10_) to the radical-trapping ubiquinol (CoQ_10_H_2_). The DHODH–ubiquinone pathway functions in mitochondria and reduces mitochondrial CoQ to ubiquinol, thereby suppressing mitochondrial lipid peroxidation (Cao J. et al., 2025); combined inhibition of DHODH and GPX4 has been shown to produce a synthetic lethal effect in tumors with high GPX4 expression (Liu Y. et al., 2026; Zhang et al., 2026). The GCH1–BH4 pathway is thought to act through both direct neutralization of lipid peroxides and inhibition of pro-ferroptotic arachidonate lipoxygenases (ALOXs). In triple-negative breast cancer, low GPX4 expression has been associated with a drug-tolerant persister phenotype driven by adaptive upregulation of FSP1, representing a form of acquired resistance.

Transcriptional reprogramming also contributes to resistance. NRF2, a transcription factor involved in antioxidant defense, can bind to promoters of ferroptosis-suppressing genes including GPX4 and SLC7A11, leading to their upregulation and contributing to resistance against GPX4 inhibitors in several cancers (Chen ZW. et al., 2025).

Aberrant post-translational modifications have also been implicated. Palmitoylation mediated by ZDHHC8, methylation by PRMT5, and deubiquitination by OTUD4 have all been reported to enhance GPX4 stability and reduce sensitivity to GPX4 inhibitors (Huang et al., 2025; Zhou L. et al., 2025; Fan et al., 2025).

The tumor microenvironment represents another source of resistance. Studies have shown that tumor-associated macrophages and their exosomes can regulate GPX4 subcellular localization and function through PRDX6, thereby contributing to ferroptosis resistance (Hu et al., 2025a).

Responses to GPX4-targeted therapy also vary considerably across tumor types and genetic backgrounds. Pancreatic ductal adenocarcinoma, clear-cell renal cell carcinoma, and thyroid cancer have been reported to be relatively sensitive to GPX4 inhibitors, whereas some colorectal and non-small-cell lung cancers appear to show low intrinsic dependence on GPX4. The molecular basis for this lineage specificity is not yet fully understood (Shen et al., 2025; Lee et al., 2025). In addition, one study found that GPX4 targeting could overcome resistance to BRAF or EGFR inhibitors in colorectal cancer, although new adaptive resistance emerged with prolonged treatment (Zhao et al., 2025). Overall, current knowledge of resistance mechanisms remains incomplete, and effective strategies to reverse resistance are still lacking, which continues to limit clinical translation.

#### 7.1.5 Lack of clinical biomarker panels and insufficient evidence in non-neoplastic diseases

A reliable set of biomarkers would be useful for guiding personalized GPX4-targeted therapy, but validated candidates are currently lacking. Although GPX4 expression levels, lipid metabolic profiles, iron metabolism characteristics, and NRF2 pathway activation have been suggested as potential predictive markers, none have been confirmed in large clinical cohorts, and standardized detection criteria or response thresholds have not been established. The clinical utility of newer biomarkers, such as GPX4 post-translational modification patterns, expression of modifying enzymes, or signatures of compensatory pathways, remains to be explored, which limits the ability to predict therapeutic responses. GPX4 genetic polymorphisms, including rs713041, have been associated with disease risk, but their relevance to treatment outcome remains unclear, making patient stratification difficult at present (Wang et al., 2025b).

For non-neoplastic diseases, the clinical translational evidence base is still limited. Although numerous preclinical studies have implicated GPX4 dysregulation and ferroptosis in conditions such as myocardial ischemia–reperfusion injury, renal fibrosis, Parkinson’s disease, polycystic ovary syndrome, and osteoarthritis (Zhou et al., 2025c; Wang et al., 2025a; Ye Z. et al., 2025; Liu et al., 2025a; Shahid et al., 2025), most of these findings come from cell lines and animal models and have not been validated in large patient cohorts. Information on GPX4 expression and post-translational modifications in patient tissues, as well as their correlation with disease progression and prognosis, remains scarce. The appropriate therapeutic window, dosing, and long-term safety profile have not been defined, which hampers translation to non-oncological indications.

### 7.2 Future research directions and translational prospects

#### 7.2.1 Systematic dissection of the global regulatory network and structure–function relationship of GPX4 to identify novel precise targets

Advances in basic mechanistic understanding are likely to support further clinical translation of GPX4-targeted strategies. Future work may benefit from integrating structural biology, multi-omics approaches, and gene editing to better define GPX4 biology and its regulatory networks, which could, in turn, enable more precise targeting.

High-resolution structural information on GPX4 and its functional sites remains an area for further exploration. Building on the recently reported fin-loop structure of GPX4 implicated in neuroprotection (Lorenz et al., 2026), cryo-electron microscopy and X-ray crystallography could be used to determine structures of mitochondrial lGPX4, cytoplasmic sGPX4, nuclear GPX4, post-translationally modified variants, and interacting complexes. Such efforts may help clarify domain-specific contributions to activity, localization, and stability and could reveal allosteric sites that might be explored for drug development, potentially circumventing some limitations of active-site inhibitors.

A more comprehensive view of the GPX4 post-translational modification landscape and its crosstalk would also be valuable. Quantitative modification proteomics could help identify additional modification sites under different physiological and pathological conditions, characterize their functional consequences, and elucidate synergistic or antagonistic interactions among modifications. Single-cell sequencing may further clarify heterogeneity across tissues and cell types and could aid in distinguishing tumor from normal cell regulation, with the potential to identify tumor-specific modifying enzymes for more selective intervention (Huang et al., 2025; Zhou L. et al., 2025; Fan et al., 2025; Zhou et al., 2025c).

In addition, expanding the understanding of non-canonical GPX4 functions may open new avenues. The immunomodulatory roles of extracellular GPX4, as well as its non-ferroptotic functions in genome stability, stem cell homeostasis, senescence, and metabolic reprogramming, warrant systematic investigation. Such studies could broaden the range of disease contexts in which GPX4 modulation may be relevant and may uncover additional therapeutic targets (Liu J. et al., 2026; Hu et al., 2025b).

To translate the opposing roles of GPX4 across diseases and cell types into actionable guidance, we propose a two-axis framework based on disease context and cell-type specificity (Table 6). In malignancies where GPX4 supports survival and resistance, inhibition is justified; in degenerative conditions, acute injuries, and fibrosis where GPX4 loss drives ferroptotic damage, stabilization is preferred. Cell-type specificity further refines this distinction: GPX4 loss in tumor cells may be therapeutic, whereas loss in CD8^+^ T cells could impair antitumor immunity, and loss in renal epithelia or neurons exacerbates tissue injury. This matrix is intended to guide preclinical strategy design and highlight situations where cell-type-specific delivery or combination regimens are particularly critical.

**TABLE 6:** Decision framework for GPX4-targeted intervention across disease contexts.

Disease context	GPX4 status/role	Intervention direction	Rationale and key cell types	Critical considerations
Solid tumors (lung, breast, thyroid, liver, and ovarian)	Overexpressed; suppresses ferroptosis; drives survival, metastasis, and therapy resistance	Inhibition	GPX4 blockade induces ferroptosis in malignant cells; enhances chemo-/radiosensitivity (Yuan et al., 2022; Liu et al., 2021a; Zhao et al., 2023a; Wu et al., 2022a; Sekhar et al., 2022; Almahi et al., 2022)	Acquired resistance *via* FSP1/DHODH upregulation (Mao et al., 2021; Cao et al., 2025b; Liu et al., 2026b; Zhang et al., 2026); rational combinations needed (Shin et al., 2018; Mao et al., 2021)
Neurodegeneration (PD, MS, and schizophrenia)	Downregulated or dysfunctional; loss promotes neuronal ferroptosis	Activation/stabilization	Restoring GPX4 protects neurons from oxidative damage and ferroptotic death (Zhang et al., 2024; Schriever et al., 2017; Liu et al., 2024b)	Blood–brain barrier penetration required (Shahid et al., 2025); long-term safety in non-neuronal tissues remains unknown
Ischemia–reperfusion injury (kidney and heart)	Reduced by degradation (e.g., TRIM21/OTUD5 axes); ferroptosis drives cell death and organ failure	Activation/stabilization	Preserving GPX4 in renal tubular epithelia and cardiomyocytes limits damage and preserves function (Sun et al., 2023; Liu et al., 2023; Chu et al., 2023; Wang et al., 2025a)	Timing is critical; delayed activation may be ineffective; systemic effects of E3/DUB modulators remain unclear (Sun et al., 2023; Chu et al., 2023)
Autoimmunity/SLE	Transcriptional repression in neutrophils triggers ferroptosis and NETosis, driving pathology	Activation	Preventing GPX4 loss in neutrophils suppresses autoimmune initiation and progression (Li et al., 2021a)	Cell-type-specific delivery to neutrophils is challenging; broad activation may affect other immune subsets
Fibrotic diseases (renal, liver, and pulmonary)	Downregulation promotes epithelial ferroptosis, which drives fibrotic signaling and matrix deposition	Activation/stabilization	GPX4 upregulation attenuates epithelial ferroptosis and subsequent fibrosis (Zhang et al., 2022; Zhang et al., 2023; Bi et al., 2024; Tsubouchi et al., 2019; Liu et al., 2025a)	Chronic administration may be required; off-target effects on fibroblasts or myofibroblasts need evaluation
Immunotherapy context	Sustains CD8^+^ T-cell function; extracellular GPX4 suppresses DC-mediated antitumor immunity	Context-dependent	In CD8^+^ T cells, preservation is beneficial (Chen et al., 2024b); in tumor cells, inhibition is desired (Liu et al., 2025a)	Cell-type-selective targeting is critical; systemic inhibition may impair antitumor immunity (Chen et al., 2024b); extracellular GPX4 neutralization may enhance immunotherapy (Liu et al., 2025a)

#### 7.2.2 Development of next-generation highly druggable and specific GPX4-targeted chemical entities

To address current druggability limitations, future development of GPX4-targeted agents may benefit from structure-based drug design and medicinal chemistry optimization.

Structure-guided rational design could facilitate the development of novel small-molecule modulators. Targeting the active site, allosteric sites, or protein–protein interfaces may yield non-covalent inhibitors or activators with improved selectivity, water solubility, and metabolic stability, potentially overcoming the off-target toxicity and poor pharmacokinetic properties associated with classical covalent inhibitors. Isoform-selective GPX4 inhibitors could be explored for cancer indications, while GPX4 activators or stabilizers, such as optimized noscapine derivatives (e.g., compound 428), may have potential in ischemia–reperfusion injury and neurodegenerative diseases (Liu et al., 2025b; Zhang et al., 2025a; Qian et al., 2025a).

Next-generation targeted protein degradation technologies also warrant further optimization. Developing GPX4-directed PROTACs, AUTACs, and molecular glues with enhanced membrane permeability, *in vivo* stability, and favorable pharmacokinetic profiles remains an area of active interest. Tumor microenvironment-responsive degrader prodrugs designed for site-specific activation could help reduce systemic toxicity and support preclinical evaluation (Yang et al., 2025; Gong et al., 2025).

Dual- or multi-targeted strategies may offer another avenue to address compensatory resistance. Designing agents that simultaneously inhibit GPX4 and parallel pathways such as FSP1, DHODH, or GCH1 could help block compensatory ferroptosis defense mechanisms. Combining GPX4 inhibition with immune checkpoint blockade or oncogene targeting might also be explored to enhance anti-tumor immunity. Further optimization of dual PCBP1/2-GPX4 inhibitors and other multi-targeted molecules may improve their *in vivo* activity and druggability for use in resistant tumors (Yu et al., 2025; Cao et al., 2025a).

#### 7.2.3 Establishment of tissue-/cell-specific delivery systems to resolve safety and efficacy conflicts

Precision delivery strategies are likely to be important for reducing systemic toxicity and facilitating clinical translation of GPX4-targeted therapies. Multi-dimensional delivery systems designed to target specific tissues, cell types, or subcellular compartments may help improve the selectivity of intervention.

For tumor-specific delivery, antibody–drug conjugates (ADCs) targeting tumor-associated antigens and tumor membrane-targeting nanocarriers have been explored as possible approaches. pH-, ROS-, or hypoxia-responsive nano-prodrugs, such as mano-PDTACs, have been developed to enable tumor-selective accumulation and activation, with the aim of minimizing off-target exposure and systemic toxicity.

At the organelle and cell-type levels, delivery systems that target the mitochondria or the nucleus have been investigated, including mitochondria-penetrating peptide-GPX4 inhibitor conjugates (Xiao et al., 2025). Cell-type-specific delivery to macrophages, renal tubular cells, or neurons has also been pursued using cell-specific promoters, targeting peptides, or exosome-based carriers, which may help address the opposing therapeutic needs in different disease contexts (Zhang et al., 2025b).

In addition, delivery systems capable of crossing biological barriers are an area of ongoing development. Liposomes, exosomes, and cell-penetrating peptide conjugates have been studied for their ability to cross the blood–brain barrier, with potential applications in Parkinson’s disease, stroke, and spinal cord injury (Shahid et al., 2025; Wei et al., 2025). Nanocarriers designed for deep penetration into solid tumors may also help improve drug accumulation and overcome physical delivery barriers.

#### 7.2.4 Elucidating the global resistance landscape and establishing rational combination therapies

Future studies may benefit from a systematic characterization of resistance mechanisms associated with GPX4-targeted therapy, which could guide the design of combinatorial strategies to improve efficacy.

To map the resistance landscape, approaches such as genome-wide CRISPR screening, single-cell multi-omics, and patient-derived organoid models could be used to identify primary and acquired resistance mechanisms, as well as potential biomarkers and molecular signatures that may inform combination regimens (Zhao et al., 2025).

Several combinatorial strategies are worth exploring. Combining GPX4 inhibitors with inhibitors of compensatory pathways such as FSP1, DHODH, or GCH1 may help counteract resistance. The concurrent use of post-translational modification regulators, including ZDHHC8 or PRMT5 inhibitors, could potentially block GPX4 stabilization (Huang et al., 2025; Zhou L. et al., 2025; Fan et al., 2025). GPX4 inhibition might also be combined with chemotherapy, radiotherapy, or other targeted agents to overcome conventional treatment resistance (Zhao et al., 2025). In the context of immunotherapy, co-administration with anti-PD-1 or anti-PD-L1 antibodies may interfere with the extracellular GPX4-ZP3 immunosuppressive axis and support ferroptosis-driven anti-tumor immunity (Liu J. et al., 2026). In addition, combining GPX4 targeting with metabolic interventions that affect glutamine or cystine metabolism may enhance ferroptosis sensitivity (Shen et al., 2025; Baskar, 2025).

#### 7.2.5 Establishing a multi-dimensional biomarker system for precision personalized therapy

Multi-omics approaches and clinical cohort studies may help establish a panel of biomarkers that could be used for patient stratification and personalized GPX4-targeted therapy. To identify and validate predictive biomarkers, large-scale clinical cohorts could be profiled using genomic, transcriptomic, proteomic, modification proteomic, and metabolomic analyses. Candidate markers such as GPX4 polymorphisms, NRF2 pathway mutations, ferroptosis-related gene signatures, GPX4 modification status, and lipid or iron metabolic profiles would require validation with standardized assays and defined response thresholds before they could be applied clinically (Chen ZW. et al., 2025; Wang et al., 2025b). In addition, *in vitro* drug response prediction models could be developed using patient-derived organoids and xenografts to create personalized screening platforms. Non-invasive dynamic monitoring might also be achieved through analysis of circulating tumor cells, exosomes, or other biofluid markers, allowing tracking of GPX4 expression, modification patterns, and treatment responses over time. Such approaches may allow real-time adjustment of therapeutic strategies and early detection of resistance.

#### 7.2.6 Expanding translational applications of GPX4 targeting in non-neoplastic diseases

GPX4 modulation may also have potential applications beyond oncology, although systematic preclinical evaluation and mechanistic studies are needed to fill current evidence gaps and clarify its therapeutic scope in non-cancer indications.

In cardiovascular diseases, agents targeting the STING–GPX4 axis have been explored to stabilize GPX4 and inhibit myocardial ferroptosis, with possible relevance to ischemia–reperfusion injury and heart failure (Wang et al., 2025a; Shi et al., 2025). In kidney diseases, approaches directed at the Smad3–GPX4 and Nrf2–GPX4 axes have been investigated to suppress renal tubular ferroptosis and attenuate fibrosis (Liu et al., 2025a; Cui et al., 2025). For neurodegenerative conditions, blood–brain barrier-penetrant GPX4 activators or stabilizers have been explored for their potential to inhibit neuronal ferroptosis and improve outcomes in Parkinson’s disease, Alzheimer’s disease (AD), stroke, and spinal cord injury (Lorenz et al., 2026; Shahid et al., 2025; Wei et al., 2025). In glioma, the natural compound paeonol has been shown to induce ferroptosis by depleting lipid droplets and increasing lipid peroxides and reactive oxygen species, leading to suppressed proliferation and metastasis, presenting a potential strategy against temozolomide resistance (Li et al., 2026). In AD, ferroptosis has been implicated in cognitive decline through pathways involving GPX4, HIF1α, autophagy, and mTOR signaling, and ferroptosis inhibitors or iron chelation have shown promise in preclinical models (Khan et al., 2025). These findings highlight the expanding therapeutic scope of ferroptosis modulation in central nervous system disorders. In reproductive medicine, GPX4 modulators have been studied for their effects on oxidative damage and ferroptosis in granulosa and germ cells, with potential implications for polycystic ovary syndrome, premature ovarian failure, and male infertility (Ye Z. et al., 2025; Tian et al., 2025; Ke et al., 2025).

In addition, further investigation of GPX4 function in organ fibrosis, aging-related disorders, and infectious diseases may broaden the range of conditions in which GPX4-targeted interventions could be relevant (Hu et al., 2025b; Chen F. et al., 2025). For most of these non-oncological indications, the therapeutic rationale remains at an exploratory stage, supported by preclinical evidence that has not yet been translated into clinical proof-of-concept studies.
